# Detection and quantification of DNA double-strand breaks by genomic and imaging methods: a critical appraisal of capabilities and limitations

**DOI:** 10.1093/nar/gkag1035

**Published:** 2026-11-04

**Authors:** Jurek Dobrucki, Maga Rowicka

**Affiliations:** Department of Cell Biophysics, Faculty of Biochemistry, Biophysics, and Biotechnology, Jagiellonian University, Gronostajowa 7, 31-326 Kraków, Poland; Department of Biochemistry and Molecular Biology, University of Texas Medical Branch, Galveston, TX 77555, United States

## Abstract

DNA double-strand breaks (DSBs) are the most genotoxic form of DNA damage. Unrepaired DSBs may cause loss of chromosome fragments at mitosis, while incorrectly repaired DSBs can cause chromosome aberrations and other adverse effects, leading to cell cycle arrest, senescence, cell death, or neoplastic transformation. Detecting and quantifying DSBs is important in studies of genome instability, aging, and cancer. Methods of detecting and quantifying DSBs fall into two broad categories: imaging-based methods, used to detect and localize DNA breaks in individual cells, and genomic methods, a new category utilizing massively parallel DNA sequencing to map DSBs genome-wide in a cell population. Imaging and genomic methods yield fundamentally different information. This review focuses on a critical evaluation of the capabilities and limitations of genomic and imaging methods for the detection and quantification of DSBs. It explains why the class of genomic methods, where quantification is based on counting the detected unique DSBs and the number of cells in the sample, results in inaccurate assessment of the average number of DSBs per cell and how this problem can be solved. We also discuss complementarity of genomic and imaging methods and potential directions of their future development.

## Graphical Abstract

**Graphical Abstract:**
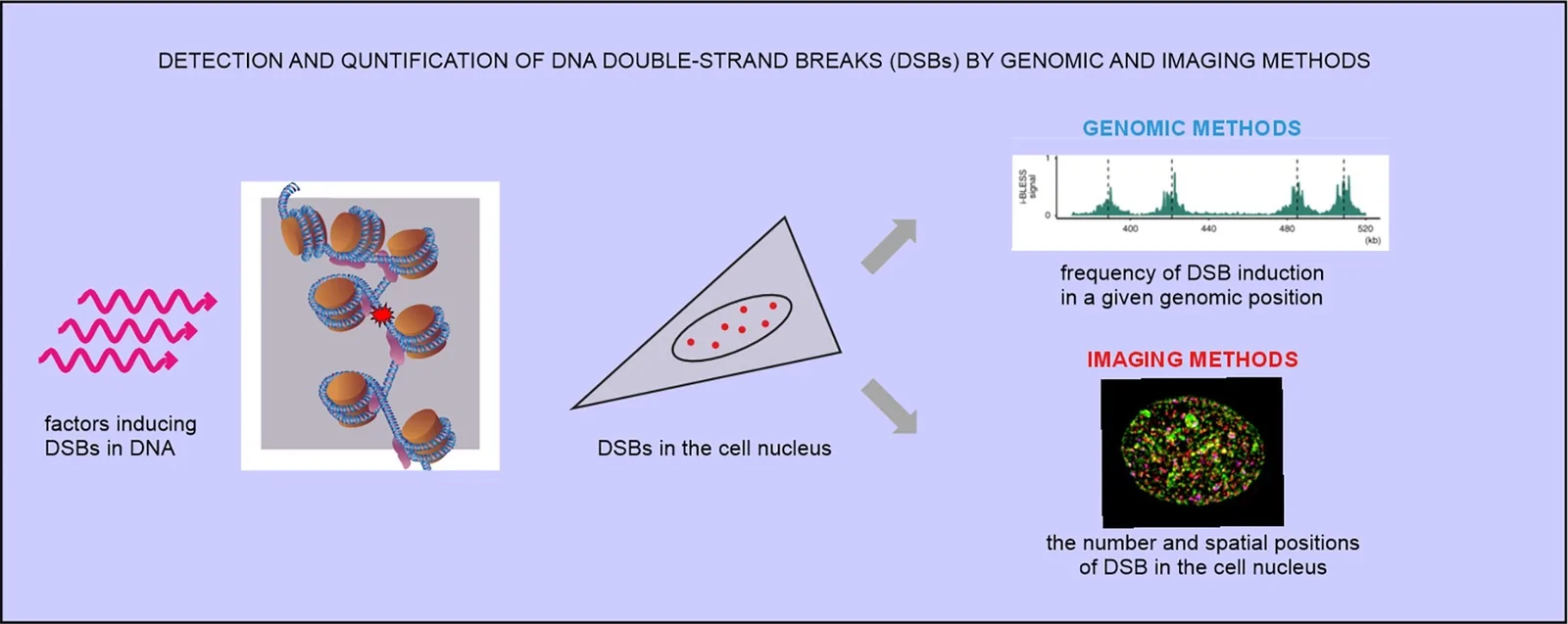

## Introduction

The most lethal type of damage is a DNA double-strand break (DSB), where phosphodiester bonds in both complementary strands are broken. DSBs can be induced by endogenous factors, such as mitochondrial reactive oxygen species or replication stress [1–4], and exogenous factors, such as exposure to genotoxic chemical agents (e.g. chemotherapeutic drugs) or ionizing radiation [5–7]. Unrepaired DSBs can cause genome instability and lead to neoplastic transformation or cell death [8, 9]. Controlled induction of DSBs is also part of increasingly popular genome-editing methods [10]. Therefore, detecting and quantifying DSBs is important in studies of the mechanisms of genomic instability, aging, cancer onset and progression, the effects of chemotherapeutics or ionizing radiation on normal and tumour cells, the off-target effects of gene-editing methods, and many other areas of biomedical research.

DSBs can be detected and quantified by approaches that are either genomic (based on massively parallel DNA sequencing) [11, 12] or imaging (based on fluorescence microscopy) [13–15]. Importantly, genomic and imaging methods for DSB detection yield fundamentally different information. Sequencing provides single-nucleotide resolution maps of the relative genome-wide frequency of DSBs in a cell population, while imaging provides estimates of the total number of DSBs in individual cells (Fig. 1). In both approaches, the initial step of the detection procedure is similar and relies on labelling or tagging DNA ends at a break. Eventually, however, genomic and imaging techniques differ in the methods of signal detection, amplification, and data analysis, as discussed below.

**Figure 1.**
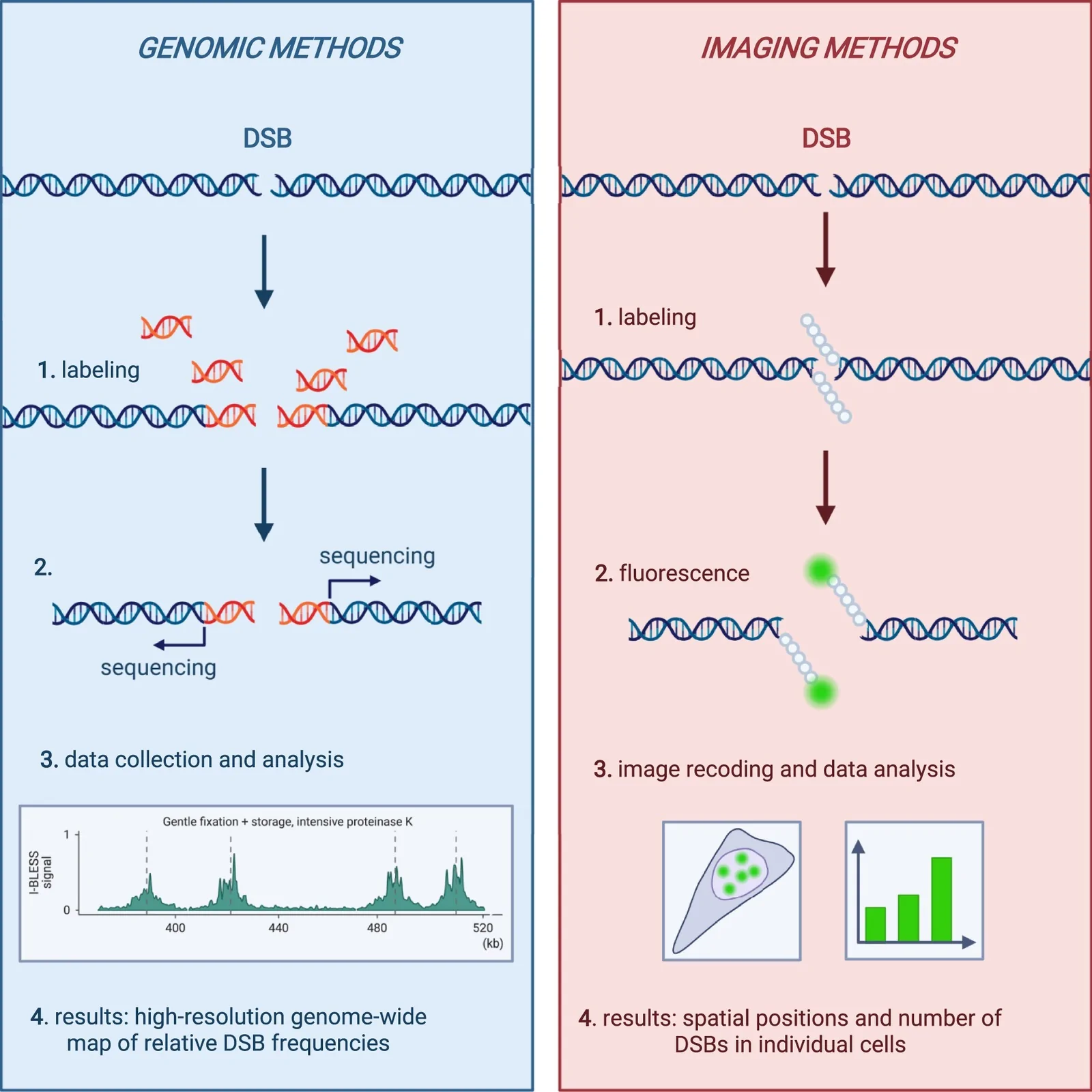
Overview of DSB detection by genomic and imaging methods. LEFT: Genomic methods of DSB detection (blue background). Following induction of DSBs, DNA ends are labelled (Stage 1) with short oligonucleotides (typically Illumina adapters). These ligated oligonucleotides are sequenced alongside the DNA ends originating from the DSBs (Stage 2). Subsequently, these molecular barcodes enable the identification of DSBs during data collection and analysis (Stage 3). In modern genomic workflows where traditional barcodes are substituted with Illumina adapters, only the fragments originating from DSBs are amplified in the flow cell and subsequently sequenced. Ultimately, genomic methods yield a high-resolution map of the relative frequencies of DSBs at each genomic position, which represents averages for the whole cell population. RIGHT: imaging methods (red background). In imaging methods, a primary and fluorescent secondary antibody, nucleotide analogues (the grey fragments), or a repair factor tagged with a fluorescent protein binds to the DNA end. This allows detection of the DSB ends in individual live or fixed cells by microscopy. Although the initial labelling step in imaging methods is conceptually similar to genomic workflows, imaging methods provide fundamentally distinct information by detecting the number of DSBs in individual cells and their spatial locations within the 3D structure of the cell nucleus, rather than population-wide DSB frequencies throughout the genome obtained from genomic methods (Created in BioRender. Dobrucki, J. (2027) https://BioRender.com/xr4fwrc).

Current DSB detection methods, both genomic and imagining, can be classified as direct (that is, employing a direct reaction with a DNA end) orindirect (dependent on DNA damage response (DDR)), as described in the next section (Figs 2 and 3).

**Figure 2.**
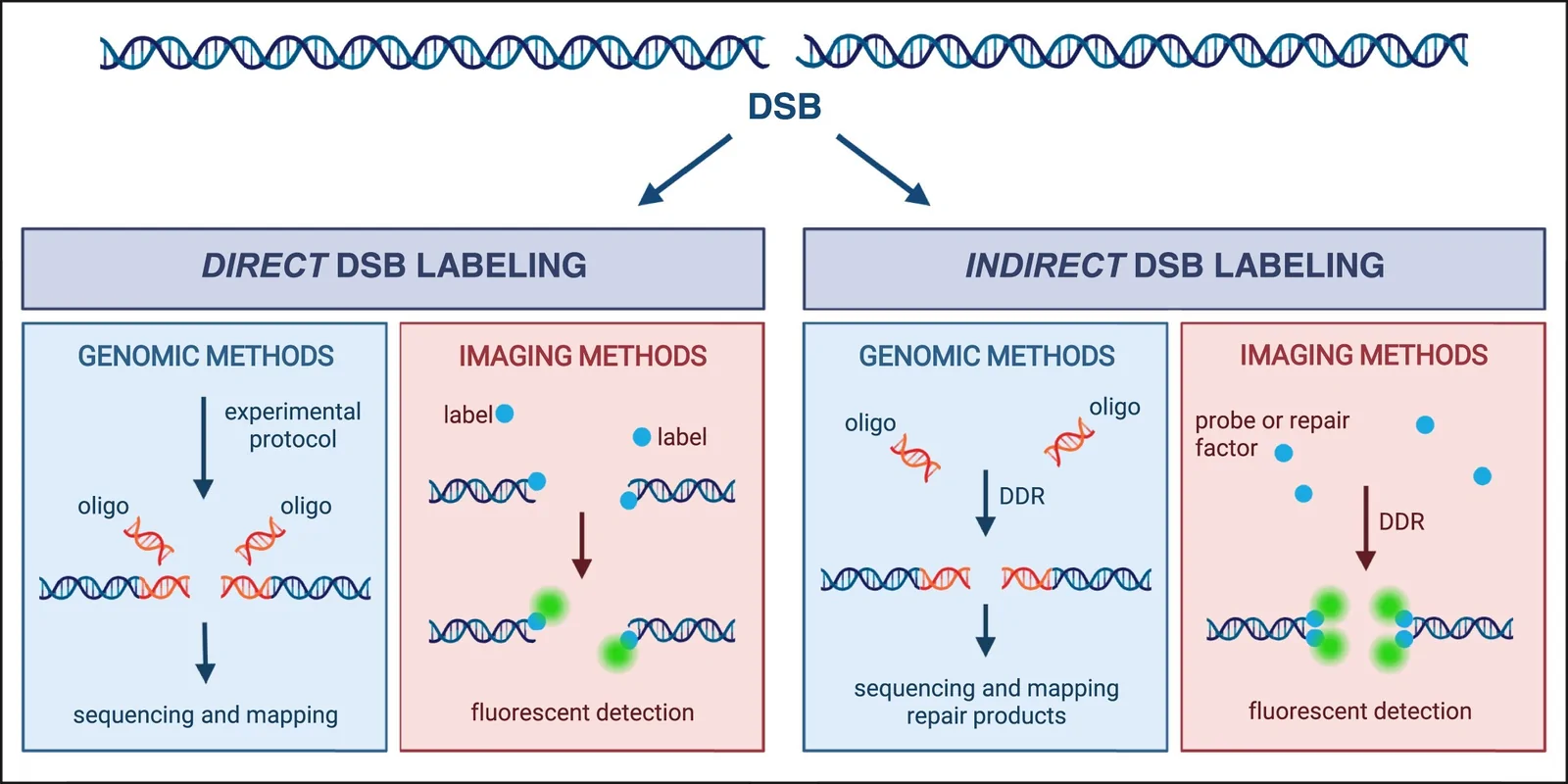
Comparison of the underlying principles of direct and indirect DSB detection methods. Direct methods rely on direct reaction with a DNA end at a break site, leading to the attachment of an oligo (in genomic methods) or a label (in imaging methods). In the subsequent steps of the procedure, oligos are used to amplify DNA fragments originating from free DNA ends (DSBs), which are later mapped to a genome, while labels are used to attach fluorescent molecules and detect the breaks in cells *in situ* by microscopy. Indirect methods rely on active DDR to attach an oligo, a probe, or a repair factor to a DSB in a living cell. In this sense, these methods detect repair events rather than the DSBs themselves. In imaging methods, the attached molecules are fluorescent or are subsequently bound by fluorescent molecules, allowing imaging of a DSB by microscopy (Created in BioRender. Dobrucki, J. (2027) https://BioRender.com/8qlmmrz).

**Figure 3.**
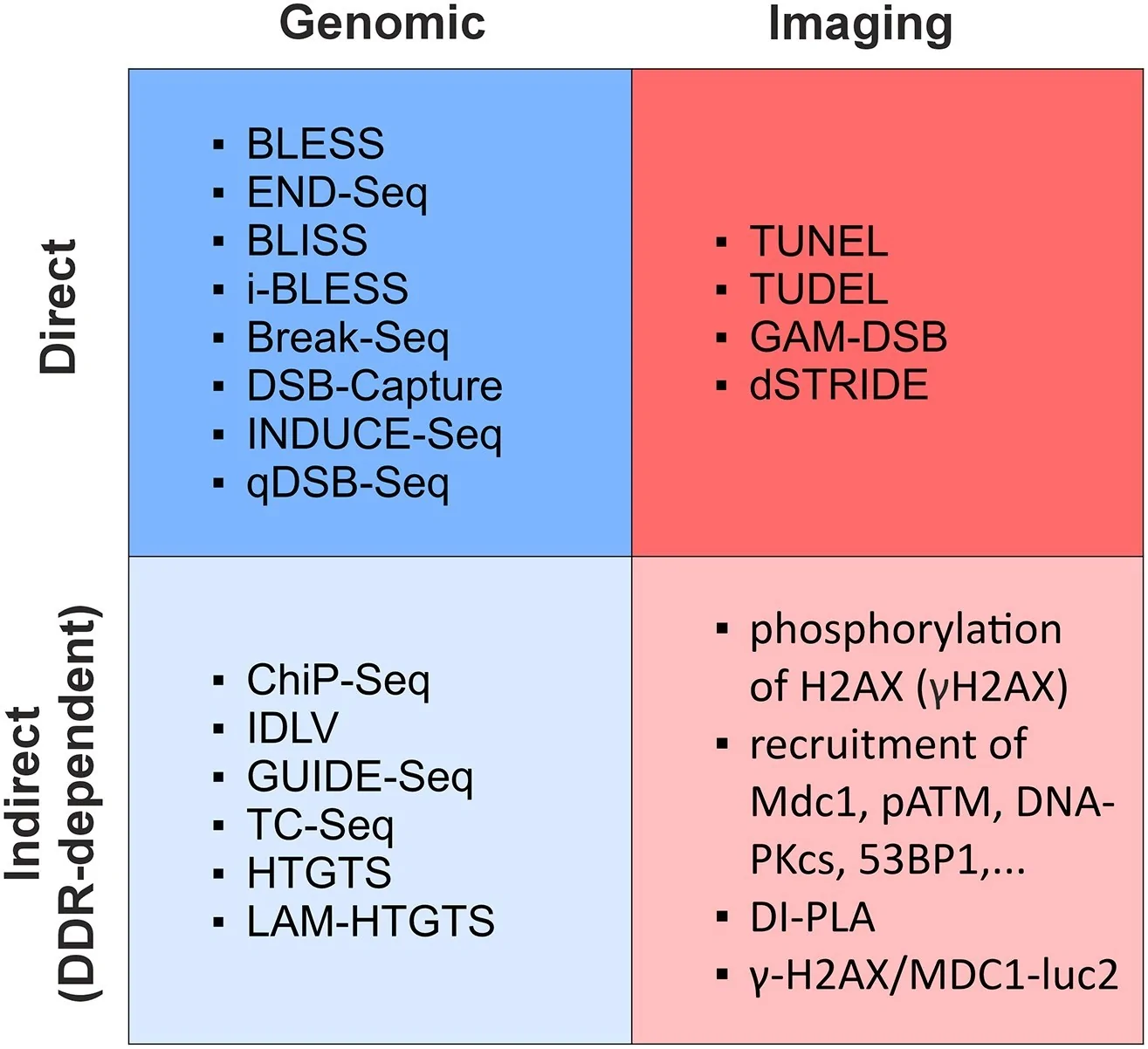
Examples of genomic (blue background) and imaging (red background) methods for direct (direct reaction with a DNA end) or indirect (DDR-dependent) detection of DSBs. Direct genomic methods rely on attaching a molecule to a DNA end at the breakage site that will be used to selectively amplify the reads originating from DSBs and then to map them to the genome or to convert it to a fluorescence signal and image by microscopy. Indirect methods rely on DDR to achieve these goals. Other DDR proteins that are recruited to DSBs, including 53BP1, Rad51, or Ku, also form repair foci but may not be suitable for the quantitative analysis of DSBs, as discussed in [13]. Table 1 lists these methods and provides a brief explanation of their principles.

## Direct and indirect DSB detection methods

Direct detection of DSBs in genomic methods relies on attaching an oligonucleotide to a DNA end by an enzyme (ligase). In the last decade, massively parallel DNA sequencing on the Illumina platform was employed to detect DSBs by ligating oligos with specific sequence(s) at a break site (Fig. 2, left panel, blue background, and Fig. 3). This sequence is later used to selectively enrich reads originating from DSBs and is removed before reads are mapped to the genome to infer genomic localization of DSBs. In the early methods, in-house barcodes were used for that purpose [12]; in newer methods, Illumina adapters, which had to be used anyway to prepare sequencing libraries, are used for selective amplification of reads originating directly from DSBs prior to sequencing. Direct detection of a DSB using microscopy can be achieved by a chemical reaction of a low-molecular-weight molecule (nucleotide analogue or oligonucleotide) with a DNA end (Fig. 2, left panel, red background). This reaction can be catalysed by terminal deoxynucleotidyl transferase (TdT), as in the TdT dUTP nick end labelling (TUNEL) assay [16, 17], TdT-dUTP DSB end labelling (TUDEL) [18], and sensitive recognition of individual DNA ends (dSTRIDE) [19], or ligase, as in DNA damage *in situ* ligation followed by proximity ligation (DI-PLA) assay [20]. The attached molecules are subsequently used to generate a fluorescent signal by immunofluorescence (as in TUNEL) or a modification of proximity ligation assay (PLA) (as in dSTRIDE).

Inindirect genomic approaches (Fig. 2, right panel, blue background, and Fig. 3), massively parallel DNA sequencing can be used to detect DDR factors, e.g. using chromatin immunoprecipitation sequencing (ChIP-seq) [21–24] against the same proteins that are visualized by indirect imaging methods (e.g. ChIP-seq against γH2AX) [25]. Moreover, DSB repair via non-homologous end joining (NHEJ) can be harnessed to label DNA breaks. Namely, the NHEJ machinery can be utilized to label DSBs by incorporating (a) short double-stranded DNA (dsDNA) oligos (GUIDE-seq [26]) or (b) integration-deficient lentiviral vectors (IDLVs [27]) at the DSB sites, or (c) by generating translocation junctions between emerging DSB ends and a bait DSB, (TC-Seq [28], HTGTS [29], or (LAM)-HTGTS [29, 30]). Subsequently, DSB positions are inferred from analysis and mapping of sequencing reads originating from fragments containing NHEJ-induced junctions. More information about these techniques is given below.

Indirect detection of DSB in imaging approaches (Fig. 2, right panel, red background and Fig. 3) employs cellular reactions to a DNA break occurring in live cells, such as histone phosphorylation (histone H2AX phosphorylated on serine 139) [15, 31] or recruitment of MDC1, pATM, DNA-PKcs, 53BP1 [32–37], and others repair factors. The presence of these post-translational modifications or accumulated factors (or both, as in DI-PLA [20] and γ-H2AX/MDC1-luc2 [38]) is translated into detectable fluorescence signals by immunofluorescence or imaging of FP-tagged proteins. This way, the localization and the number of DSBs in individual cells can be estimated.

Basic information on the principles and capabilities of the major genomic and imaging methods of detecting and quantifying DSBs is summarized in Table 1.

**Table 1.** Major genomic and imaging methods developed for DSB detection, including a brief description of their underlying principles. The methods are listed in a chronological order of publication.

	Abbreviation	Principle	Ref.
**GENOMIC**			
Direct	BLESS	The first genome-wide mapping technique of DNA DSBs at nucleotide resolution in fixed cells. The approach utilizes biotinylated hairpin oligonucleotide linkers that are ligated directly to the exposed DNA ends *in situ* within fixed nuclei. Following genomic DNA extraction and fragmentation, the labelled break sites are selectively captured using streptavidin-biotin system, allowing the fragments containing the original DSBs to be enriched, sequenced, and precisely mapped to the genome.	[12]
	Break-seq	Developed as an alternative *in situ* mapping approach, this method adapts the core principles of BLESS by embedding live cells in agarose beads prior to cell lysis. Following embedding, DNA double-strand ends are blunted using T4 DNA polymerase (and ligated to biotinylated adapters). However, due to the inherent enzymatic properties of the blunting phase, this formulation exhibits a preferential labelling bias towards 5′ DNA overhangs [39, 40]. Updated version of the procedure [41] includes innovative 3D printing of agarose plugs, but still relies on T4 polymerase, maintaining bias towards labelling 5′-overhang ends.	[41, 42]
	END-Seq	Building upon the established principles of genome-wide, single-nucleotide resolution mapping, END-Seq substantially improved sensitivity of DSB detection. This was achieved by avoiding fixation-induced artifacts by skipping that step and by embedding cells within agarose plugs to minimize mechanical shearing. Another innovation was using exonuclease to blunt DSB ends. END-seq has a sensitivity of detecting at least one DSB per cell among 10 000 cells not harbouring DSBs.	[43, 44]
	DSBCapture	This method maps DSBs at single-nucleotide resolution by integrating the *in situ* break-labelling step with Illumina library preparation to streamline the procedure and reduce artifacts. Building upon the BLESS framework, it replaces blunt-end ligation with a cohesive-end ligation using a modified, T-tailed P5 Illumina adapter directly at the break site, not as a second ligation. This single-step adapter integration eliminates downstream library preparation steps and eliminates the need for low-sequence-diversity protocols [45].	[46]
	BLISS	This method allows the *in situ* DSB labelling in low-input samples by utilizing unique molecular identifiers (UMIs) to track individual DSBs. Performed on fixed cells or tissue sections adhered to a glass slide to prevent centrifugal shearing, the protocol ligates adapters containing short random UMIs to blunted DNA ends prior to genomic DNA extraction and linear amplification via T7-mediated *in vitro* transcription. While this framework represents a distinct conceptual innovation that radically lowers the amount of required starting material, practical limitations led to the newer version of the protocol going back to in-suspension labelling of DSBs (in suspension BLISS, or sBLISS).	[47]
	i-BLESS	This method, a modern version of BLESS, substantially reduces artifacts causing background noise by optimizing a degree of fixation and protein removal, while preventing mechanical shearing by immobilization in agarose beads. Furthermore, the procedure streamlines the workflow by utilizing a single-step labelling mechanism with modified Illumina adapters, integrating DSB capture directly with library construction to minimize downstream manipulation artifacts. i-BLESS has a sensitivity of detecting at least one DSB per cell among 100 000 cells not harbouring DSBs.	[40, 48]
	qDSB-seq	This modular framework is the only approach capable of providing absolute, genome-wide DSB counts per cell by introducing an endogenous spike-in control. Rather than acting as a standalone labelling chemistry, it is an analytical and experimental layer that can be integrated with any existing *in situ* DSB labelling method (including BLISS, i-BLESS, END-seq, or INDUCE-seq). Spike-in DSBs are induced at pre-determined locations using restriction enzymes and then undergo identical downstream adapter ligation and sequencing pipelines. The frequency of spike-in breaks is assessed independently using qPCR or sequencing, and the ratio of sample reads to spike-in reads allows for direct calibration, overcoming the capturing and amplification inefficiencies of the underlying protocol to yield true absolute DSB quantification.	[49]
	sBLISS	In-suspension BLISS, an improved version of the BLISS protocol, where DSB labelling is performed in suspension, not on glass slides.	[50]
	INDUCE-Seq	The only technique of detecting DSB via sequencing not utilizing PCR amplification of the sample and thus avoiding PCR bias. Details of the technique are proprietary, but its core labelling protocol is very similar to BLISS, although no UMIs are used.	[51]
Indirect	ChIP-seq	This method detects DSBs indirectly by combining sequencing with chromatin immunoprecipitation against chromatin-associated proteins or histone modifications that accumulate at break sites. Following cell fixation and chromatin fragmentation, antibodies are used to immunoprecipitate specific protein markers of the DDR (such as γH2A.X or 53BP1). The associated DNA is then purified and sequenced. Because this approach relies on the biological recruitment of protein proxies across a surrounding chromatin domain, it cannot identify exact DNA breakpoints and reflects cellular signalling activity rather than physical DNA strand cleavage.	[21–24]
	IDLV	This indirect method maps *in vivo* DSBs by exploiting the cell’s natural non-homologous end-joining (NHEJ) pathway to capture and integrate episomal viral genomes into active break sites. Cells are transduced with an integrase-defective lentiviral vector, and the resulting linear, double-stranded viral DNA genomes present in the nucleus act as physical traps that are ligated directly into transient genomic DSBs during the cellular repair process. The stable integration sites are subsequently identified via sequencing, and clustered integration site (CLIS) analysis is used to computationally distinguish nuclease-induced breaks from background, naturally occurring DSBs. Because this approach relies entirely on the successful transfection, nuclear entry, and cellular NHEJ-mediated integration of a large exogenous viral cassette, it only captures breaks repaired via the NHEJ pathway and is limited by viral delivery efficiencies across different cell types.	[27]
	TC-Seq	This indirect method maps genomic DSBs by capturing and sequencing chromosomal rearrangement events joined to an engineered, endonuclease-induced ‘bait’ locus. Utilizing cells genetically modified to harbour an I-SceI meganuclease target sequence at a specific locus (e.g. *c-myc* or *IgH*), the protocol induces a targeted DSB to bait translocations. Following genomic DNA extraction and mechanical fragmentation, asymmetric DNA linkers are ligated to the ends, and rearrangements joined to the bait locus are amplified via semi-nested ligation-mediated PCR. A distinct technical advantage of this method is that mechanical sonication generates unique linker ligation points for each fragment, allowing independent translocation events to be accurately clustered and distinguished without requiring sequencing through the actual rearrangement breakpoints. However, because it relies on the biological completion of a translocation event to an engineered site, it exclusively maps breaks that undergo inter-chromosomal rearrangement via cellular repair pathways.	[28]
	HTGTS	This indirect mapping approach identifies genome-wide DSBs by capturing chromosomal translocation junctions that form between naturally or synthetically occurring breaks and a fixed ‘bait’ DSB. Similar to TC-Seq, the method relies on cellular end-joining pathways to fuse a known, endonuclease-cleaved bait locus (such as an engineered I-SceI site in the *c-myc* or *IgH* gene) to other active DSBs across the genome. Following genomic extraction and fragmentation, linear PCR using a biotinylated primer specific to the bait locus isolates the target junctions, which are then adapted and amplified for high-throughput sequencing. While highly sensitive for identifying recurring oncogenic rearrangements and nuclease off-target sites, HTGTS is fundamentally a translocation assay; it exclusively detects the subset of genomic breaks that physically interact with, and translocate to, the chosen bait site before being resolved by cellular repair machinery.	[52]
	LAM-HTGTS	This method improves upon the original HTGTS framework by incorporating linear amplification-mediated PCR (LAM-PCR) and bridge adapter ligation to significantly enhance target junction yield and reproducibility. Utilizing a single 5′ biotinylated primer, LAM-PCR directly amplifies bait-prey translocation junctions from genomic DNA into single-stranded products. These fragments are enriched on streptavidin beads and stabilized via a dsDNA ‘bridge’ adapter possessing a variable 3′ overhang, maximizing ligation efficiency before subsequent nested PCR and library construction. While these technical adaptations increase junction recovery 10-to-50-fold over standard HTGTS, the approach remains an indirect translocation assay that exclusively maps the subset of genome-wide breaks that physically join to the selected bait cleavage site.	[29, 30]
	Guide-Seq	This indirect mapping method captures genome-wide DSBs by utilizing the cell’s natural end-joining machinery to integrate a blunt, chemically stabilized double-stranded oligodeoxynucleotide tag into active cleavage sites. Following tag integration, randomly sheared genomic DNA is adapted and amplified via a specialized single-tail PCR strategy that selectively sequences regions flanking the tag, avoiding the background noise and amplification bias of alternative capture methods. While highly effective for mapping nuclease off-target profiles at nucleotide resolution, its detection efficiency is fundamentally constrained by its indirect nature, as it relies completely on the successful transfection and NHEJ-mediated integration of the exogenous tag.	[26]
**IMAGING**			
Direct	TUNEL	In fixed cells, nucleotide analogues (e.g. BrdU, biotin-dUTP) are incorporated at 3′ DNA ends by TdT polymerase. Detection of the analogues at DSBs are achieved by immunofluorescence (anti-BrdU) or avidin-conjugated fluorescein or avidin-conjugated peroxidase. Due to low sensitivity TUNEL is typically used to detect extensive DNA fragmentation, such as in apoptosis, but not for detection of individual DSBs.	[16, 17]
	GAM-DSB	GAM is a protein derived from bacteriophage Mu. It binds to DNA ends at DSBs. When GFP-tagged GAM protein is expressed in transfected cells, it binds to DNA ends and reveals DNA breaks. Since GAM competes with Ku proteins for binding to DNA ends, false-negative results can occur. It can be used in live cells. Due to low sensitivity GAM-DSB assay is not suitable for efficient detection of individual DSBs.	[53]
	dSTRIDE	In fixed cells, BrdU nucleotide analogues are incorporated at 3′ DNA ends by TdT polymerase. These analogues are subsequently detected using a modified version of PLA. Enzymatic processing of dirty DNA ends is required in the procedure to detect DNA ends other than 3′. dSTRIDE can detect individual DSBs.	[19]
	TUDEL	In fixed cells, the nucleotide analogue 5-ethynyl-dUTP (EdU) is incorporated at 3′ DNA ends using TdT polymerase. Detection of the incorporated analogues is achieved by ‘click reaction’ with an azide of a fluorescent probe. It has been shown to detect lesions detected by imaging foci of γH2AX or 53BP1 in cells following exposure to ionizing radiation. An ability to detect individual endogenous breaks has not been demonstrated.	[18]
Indirect	γH2AX	In response to induction of a DSB, histone H2AX is phosphorylated on serine 139 in a large section (~ 1 Mbp) of DNA; the phosphorylated histones are detected by immunofluorescence. A widely used assay for quantitating DSBs in fixed cells *in vitro*.	[15, 31]
	DNA-PKcs recruitment	DNA-PKcs protein acts in the NHEJ pathway. It is recruited to a DSB, binds to Ku70/Ku80, and phosphorylates other proteins. PKcs in DSB repair foci can be detected by immunofluorescence.	[32, 33]
	Mdc1 recruitment	Mdc1 protein binds to γH2AX histones at a DSB. Mdc1 marking a DSB can be detected by immunofluorescence or imaging of a GFP-MDC1 fusion protein.	[34]
	pATM recruitment	ATM protein is recruited to DNA damage by the MRN complex, where it autophosphorylates and pATM phosphorylates other proteins. pATM can be detected by immunofluorescence or imaging of a GFP-tagged fusion protein.	[35]
	γ-H2AX/ MDC1-luc2 reporter	γ-H2AX/MDC1-luc2 is a genetically engineered bioluminescent reporter. N- and C- luciferase gene fragments are fused to genes encoding H2AX and Mdc1. γH2AX at a DSB interacts with Mdc1 bringing both protein fragments together, and reconstitutes luciferase activity to generate bioluminescence at a DSB. Relative levels of emission (damage) are measured.	[38]
	DI-PLA	Biotinylated dsDNA oligonucleotides are ligated at a DNA break, bound by anti-biotin antibodies, and detected by PLA by using γH2AX as the second protein required to detect the PLA signal.	[20]

## Methods of DSB detection

### Principles of labelling DSBs for genomic mapping using massively parallel DNA sequencing

In genomic methods of DSB detection, the foundational step is labelling free, dsDNA ends with oligonucleotide. Early workflows harvested naked, unprotected nuclei (e.g. BLESS) and relied on aggressive centrifugation protocols, which likely resulted in substantial, inadvertent mechanical shearing of the DNA before labelling could occur. Consequently, these early procedures labelled a high volume of artefactual background breaks arising from physical sample handling rather than the specific biological stimulus under investigation. These artefactual breaks were manifested in subsequent sequencing data as fairly homogeneous background noise across the genome that frequently obscured genuine sequence-specific DSBs of interest. This technical limitation was observed in other early methodologies like Break-seq (Fig. 4).

**Figure 4.**
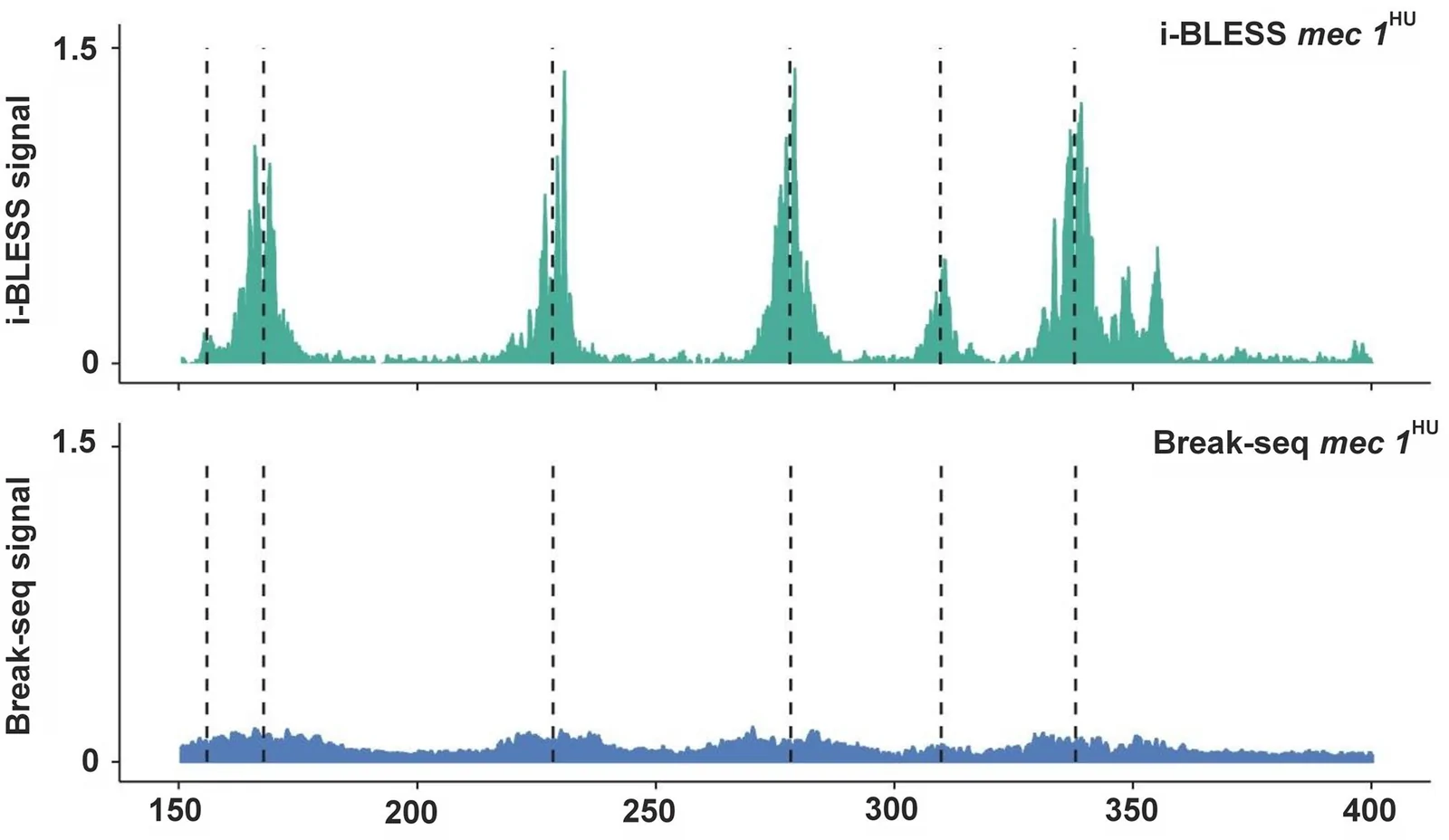
Comparison of the quality of early DSB detection via genomic methods (Break-seq) with a high-sensitivity current method (i-BLESS) [40]. The budding yeast mec1-1 sml1-1 cells were treated with hydroxyurea (HU) to induce replication stress [40, 42]. i-BLESS and Break-seq signals around early replication origins (dashed vertical lines) in a representative region of chromosome XV under HU treatment are shown. The bottom panel shows an example of an early DSB mapping method (Break-seq), which exhibited a high frequency of artefactual DSBs introduced during handling and consequently lacks well-defined peaks expected around replication origin (dashed vertical lines). Furthermore, due to the use of T4 polymerase, Break-seq is biased against detecting DSBs with 3′-overhangs, which dominate in peaks around replication origins. The top panel shows the data obtained under the same conditions, but the DSBs were mapped using a much more sensitive and more recent DSB mapping method (i-BLESS). Therefore, in i-BLESS data, DSB peaks around replication origins are clearly visible and exhibit much higher peak-to-trough ratios along the *y*-axis, indicating that the true biological signal (DSBs induced by replication stress) in this method is less obscured by background noise (artefactual DSBs occurring randomly along the genome during processing).

To eliminate these pervasive mechanical artifacts, strategies for physically safeguarding the delicate chromatin architecture were employed. By embedding intact cells in agarose prior to cell lysis, newer methods successfully insulated the genomic DNA from mechanical shearing forces. This concept was implemented using agarose plugs in END-seq and agarose beads in i-BLESS, with both methods achieving a substantial reduction in background noise.

Interestingly, although agarose immobilization was initially credited with this leap in DSB detection sensitivity—and i-BLESS was explicitly named to highlight this feature as its main advantage—a more in-depth analysis, as in [29], revealed that chemical fixation, or rather the lack thereof, was the main driver of dramatic data quality improvements (shown in greater detail in Fig. 6). In fact, Break-seq also used protective agarose plugs; nevertheless, as other early labelling techniques, it remained burdened by a high level of background noise (Fig. 4). As discussed in subsequent chapters, i-BLESS successfully demonstrates that it is possible to maintain exceptionally low noise levels even while utilizing chemical fixation, a feature that offers critical practical benefits by allowing the safe shipment of biological samples.

Another way to eliminate mechanical shearing was to fix cells or tissue sections with formaldehyde directly onto a solid surface (such as a microscope slide, coverslip, or multiwell plate), introduced in BLISS and adapted by INDUCE-seq. A more recent iteration, sBLISS, reverts to the original BLESS design of labelling DSBs in suspension while maintaining significantly reduced noise levels. Therefore, it is reasonable to conclude that the radical reduction in background noise observed across contemporary DSB sequencing methodologies (including END-seq, i-BLESS, sBLISS, and INDUCE-seq) cannot be attributed to a single overarching innovation. Instead, the highly improved sensitivity of DSB detection is the result of systematic fine-tuning of multiple individual procedural steps. Ultimately, the diverse architectures of these methods demonstrate that highly sensitive and precise DSB detection can be achieved successfully through distinct technical approaches.

The oligonucleotides utilized to label DSBs have undergone an even more profound evolution. Early methods relied on custom-designed oligonucleotide with sequences engineered for a dual purpose: first, to serve as primers for selective polymerase chain reaction (PCR) amplification, and second, to function as molecular barcodes to differentiate true DSB signals among the sequenced reads. To prevent wasteful sequencing of excessively long custom sequences, these oligonucleotides were trimmed before sequencing (e.g. to just 5–11 nt in BLESS) and frequently incorporated biotinylated hairpins that were used prior to sequencing to enrich labelled reads using streptavidin-biotin system.

As the technology matured, these workflows became highly streamlined by directly integrating with Illumina library preparation. Modern methods have largely discarded custom oligonucleotides in favour of directly ligating standard Illumina adapters. This transition relies on the realization that because amplification is routinely performed as a core component of Illumina sequencing, pre-amplifying the sample with separate primers is redundant. The INDUCE-seq technique takes this innovation to its logical conclusion: a fundamental feature of the Illumina platform is bridge amplification on the flow cell, where only fragments successfully flanked by Illumina adapters are amplified. Consequently, the flow cell itself acts as a selective enrichment mechanism for adapter-ligated reads. Capitalizing on this built-in enrichment, along with the significantly lower starting material requirements of contemporary Illumina sequencers, the creators of INDUCE-seq completely eliminated PCR amplification during library preparation prior to sequencing.

Due to the simultaneous advances in refining all aspects of the DSB labelling procedure, this single-step labelling does not result in unacceptable levels of artefactual breaks, as would have been the case in earlier methods. Nevertheless, certain advanced techniques retain multistep labelling to obtain additional data; e.g. the integration of unique molecular identifiers (UMIs) in BLISS allows for the computational removal of PCR duplicates during data analysis.

In addition to reducing noise to achieve high sensitivity in DSB detection, another key goal of contemporary methods was the ability to label diverse DNA breaks (i.e. various types of DNA ends) with uniform accuracy. Chemically modified (‘dirty’) ends, resulting from treatments by ionizing radiation, are typically ‘cleaned’ using commercially available kits prior to the DSB labelling procedure. However, existing methods vary in their ability to label the repertoire of 3′ overhangs, 5′ overhangs, and blunt DNA ends naturally occurring in cells. As mentioned earlier, methods based on T4 polymerase exhibit a strong bias towards capturing 5′ DNA ends. This makes them not suitable for studying replication-induced DSBs or the 3′ overhangs generated during the resection of stalled replication forks and homologous recombination (Fig. 4). An approach described in [54], where DSB is induced at the same locus, but with different end configurations, would be an ideal approach to more rigorously study the ability of different DSB sequencing methods to detect DSBs with different end configurations.

DSB detection methods also vary based on the specific applications they were engineered to address. While many mainstream platforms require relatively large sample sizes, such as 10 million human cells, BLISS was intentionally designed for low-input samples to enable clinical applications, particularly in oncology. INDUCE-seq similarly features remarkably low starting material requirements and was specifically engineered to eliminate PCR amplification bias. Meanwhile, i-BLESS was highly optimized for yeast; because yeast cells possess a rigid cell wall, they require different technical approaches than mammalian cells, such as upstream enzymatic wall digestion. In contrast, other approaches prioritized distinct technical milestones; for instance, END-seq focused heavily on minimizing background noise and optimizing the detection of extensive single-stranded tracks, validating its capacity to map 3′ overhangs up to 5 kb in length. This does not imply that other architectures are inherently incapable of capturing such long-range events, but rather than in alternative techniques they were not systematically benchmarked to define their upper limits in detecting such tracks. Indeed, multikilobase resection tracks were already captured and reported in the original BLESS protocol.

Ultimately, irrespective of their specific design, modern genomic mapping methods consistently deliver a single-nucleotide resolution landscape of relative DSB frequencies throughout the genome. This unprecedented sensitivity and specificity are the direct result of a decade of systematic fine-tuning, procedural streamlining, and a growing understanding of the technical artifacts and background noise that historically obscured true biological signals.

### Principles of labelling DSBs for detection by imaging-based techniques

Detection and imaging of DSBs use similar direct or indirect methods of labelling DNA ends. Direct DSB detection methods (Fig. 3), such as the TUNEL assay [17] and dSTRIDE [19], rely on adding mononucleotide analogues to the 3′ end of the DNA molecule and subsequent fluorescent labelling of the chain of the incorporated analogues in a manner that allows subsequent signal amplification. TdT (polymerase) is used in both assays. Unlike other known DNA polymerases, TdT synthesizes a new DNA molecule using only single-stranded DNA as a nucleic acid substrate. This template-independent polymerase adds random nucleotides to a 3′ end of a DNA molecule. TdT can incorporate all four nucleotides, although dGMP and dCMP are attached most efficiently. It can also incorporate various nucleotide analogues, including BrdU or biotinylated nucleotide analogues, and this capacity is utilized in the detection of DSBs by TUNEL and dSTRIDE. TdT can add nucleotides to 3′ overhang and recessed, as well as blunt, DNA ends, although with different efficiencies [55]. Two different approaches, one based on immunofluorescence (TUNEL) and the other on modified PLA assay (dSTRIDE), lead to fluorescence labelling of nucleotide analogues incorporated by TdT at DSBs and signal amplification.

Indirect imaging approaches are based on detecting cellular reaction to damage, including histone modifications (γH2AX) and recruitment of NHEJ or HR repair factors.

H2AX is a core histone variant that is phosphorylated in response to a DSB. DNA-dependent kinases phosphorylate H2AX at serine 139. This post-translational modification extends over a large region of chromatin up to a million base pairs long [31]. The phosphorylated histone H2AX (γH2AX) is detected within a few minutes after damage induction and can last for a few hours [56]. It can be detected by immunofluorescence or followed in live cells with fluorescent antigen-binding antibody fragments [57, 58] or nanobodies [59].

Several repair factors form microscopically detectable DNA repair foci, including the MRN complex, which serves as a factor sensing a DSB [14, 60], 53BP1 [61–63], DNA-PKcs [32, 33], Artemis [64], XRCC4, XLF, and DNA Ligase IV [65] that are involved in subsequent steps of NHEJ pathway, and RPA [66], Rad51 [67], and BRCA1 [68]—components of HR repair. NHEJ and HR factors mentioned earlier can be detected by immunofluorescence or imaged in cells expressing a fluorescent fusion protein.

53BP1 is frequently used to detect DSB repair by NHEJ. It is a DDR protein that binds to post-tranlationally modified histones H4 and H2A [69] and γH2AX [70, 71]. It inhibits DNA resection and HR, and promotes the NHEJ repair pathway (although the exact roles played by 53BP1 in DSB repair are still debated [72]). The recruitment of 53BP1 to DSBs is cell-cycle-dependent, and 53BP1 can form a ring surrounding a central site of active HR repair of DSBs induced by ionizing radiation [61, 63] that might represent damage clusters. It is still unclear whether 53BP1 is recruited and detectable by microscopy at each individual DSB in all phases of the cell cycle.

Since the numbers of phosphorylated H2AX histones and the numbers of recruited repair factors like 53BP1 are high, detection of DSBs that employ these methods capitalizes on this strong biological amplification. The result of an imaging experiment is an estimate of the total number of DSBs detected in each cell, or more precisely, in each imaged cell nucleus(Fig. 1), although with limitations described in the subsequent sections.

Other less widely used methods capable of detecting DSBs are DI-PLA, TUDEL, and a method that uses a γ-H2AX/MDC1-luc2 reporter. The DI-PLA assay employs PLA to detect the simultaneous presence of biotinylated nucleotide analogues incorporated at a DNA break by TdT polymerase and DDR factors—γH2AX or 53BP1 [20]. This approach ensures the specific indirect detection of a PLA-based DSB strong signal amplification. The TUDEL assay is based on the incorporation of ethinylated nucleotide analogues at the DSB by TdT polymerase and a subsequent ‘click’ reaction between these analogues and an azide of a fluorophore [18]. γ-H2AX/MDC1-luc2 is a genetically engineered bioluminescent reporter [38]. DSBs become detectable when two DDR factors, γH2AX and Mdc1, are brought into close proximity at the DNA lesion. Cells are transfected with plasmids encoding N- and C- luciferase gene fragments fused to genes encoding H2AX and Mdc1. The interaction between γH2AX and Mdc1 leads to the reconstitution of the luciferase molecule and the generation of bioluminescence at a DSB.

### Strengths and weaknesses of genomic and imaging methods for DSB detection

The defining strength of genomic approaches is their ability to map the precise relative probability of DSB induction across the entire genome. While this information is not available through imaging, the latter has the advantage of providing an estimate of the number of DSBs in individual cells and thus the range of DSB numbers/cell in the whole cell population, a type of information that is generally not available via genomic methods. A notable exception is the https://www.science.org/content/article/gene-therapy-clinical-trial-halted-cancer-risk-surfaces protocol [49], which bridges this gap by calculating the absolute average number of DSBs per cell.

Other differences include (i) the inherent blind spot of genomic methods in regard to unmappable repetitive genomic regions, and (ii) their superior ability to distinguish adjacent DSBs. While genomic DSB detection provides single-nucleotide resolution, the resolution of microscopy methods depends on the labelling technique and the imaging modality, as summarized in Table 2.

**Table 2.** Various capabilities of DSB detection by genomic and imaging methods (Y - Yes, N - No)

Able to detect	Genomic	Imaging
Chromosomal coordinates of DSBs	Y	N
DSB localization in the nucleus	N	Y
Number of DSBs in individual cells	N	Y
DSBs in large number of cells	Y	Y
Genome-wide DSB frequency	Y	N
DSB distribution across a cell population	N	Y
DSBs in regions non-mappable by sequencing	N	Y
DSBs located near each other in nucleus	Y	N

The technological trajectories of genomic and imaging methodologies are increasingly converging. Historically, these two modalities started many orders of magnitude apart in the number of cells used for analysis. Today, genomic methods strive to drastically reduce the number of starting cells, whereas imaging methods aim to massively increase the throughput of individual cells imaged to capture true population-wide data.

### Strengths and weaknesses of direct versus DDR-dependent methods of detecting DSBs

Direct and indirect DSB detection methods have important advantages and disadvantages. Direct detection is independent of cellular capacity to respond to DNA damage; therefore, DSBs can be detected even in cells that cannot phosphorylate the histone H2AX or recruit repair proteins to a DNA lesion. Some types of tumour cells have impaired capacity to repair DSBs. Therefore, in studies aimed at quantifying DNA damage in such cells, a lack of detectable cellular response will constitute a false negative. However, in indirect assays based on the detection of histone γH2AX, if histone phosphorylation were to occur in response to damage other than a DSB (as suggested in [73, 74]), this will lead to a false positive.

Using direct detection of DNA ends should help minimize false positive or false negative results at the labelling step. This constitutes a potential advantage of direct over DDR-dependent DSB detection methods. However, direct methods of DSB detection have various technical limitations and challenges of their own. For example, direct genomic methods rely on ligating oligos with specific sequences that are subsequently utilized as primers for amplification (and streptavidin-biotin enrichment, as in the BLESS method). Therefore, the inefficiency of this ligation in cells with a high presence of chemically modified (‘dirty’) ends pose a technical challenge. There is a consensus that effective detection of ‘dirty’ ends via genomic methods requires an additional step that converts ‘dirty’ ends to ends more amenable to the labelling procedure. This is resolved by employing the polynucleotide kinase 3′-phosphatase (PNK) enzyme, which converts DNA 3′-P to 3′ ends and thus creates a substrate for DNA polymerase. For example, in i-BLESS, it has been shown that for common examples (3′ versus 5′ overhangs and blunt ends), labelling efficiencies are fairly similar [40], while ‘dirty ends’ require prior repair; it is not known how more exotic end configurations would fare under DSB labelling with that method.

dSTRIDE also employs direct detection of DSBs by enzymatically catalysed (TdT) addition of nucleotide analogues to 3′ DNA ends. Other, nontypical or ‘dirty’ DNA ends remain undetected until they are converted to 3′. In the procedure currently used, this is achieved by using the T4 PNK enzyme [75]. However, the efficiency of cleaning DNA ends in the dSTRIDE procedure has not been studied systematically.

Although an ability to detect nontypical or ‘dirty’ DNA ends is particularly important when DSBs are induced by free radical reactions (such as resulting from exposure to ionizing radiation), to our knowledge, systematic studies of the efficiency of detecting nontypical or ‘dirty’ DNA ends by various genomic and imaging methods have not been carried out.

It is reasonable to assume that under typical conditions, most normal wild-type cells are able to detect various types of DNA ends, process the ‘dirty’ ends, phosphorylate histone H2AX, and recruit repair factors. Therefore, in contrast to methods that detect 3′ ends, indirect methods may have the advantage of being able to detect all types of DSBs efficiently.

Despite the use of PNK to clean DNA ends, the range of possible DNA ends (e.g. not typical as 3′ or ‘clean’) presents a challenge for both direct and indirect genomic DSB detection methods, since different types of DNA ends are unlikely to be labelled with the same efficiency. Therefore, a method of quantification based on estimations based on intentionally introduced spike-in DSBs (qDSB-Seq [40]) has the highest chance of performing such an estimation accurately, as long as the spike-in DSBs, used for normalization, have a DNA end type similar to DSBs of interest.

DDR-dependent methods of DSB detection are based on the presence of histone modifications or the recruitment of DDR proteins. However, it is important to note that the presence of these factors is not precisely coordinated with the presence of DSBs. γH2AX and 53BP1 foci become detectable minutes after the induction of damage and last beyond the completion of the repair (the exact duration of this time is likely to vary) [76]. This lack of coordination in time may contribute to the fact that some γH2AX and 53BP1 foci do not co-localize in cells challenged with genotoxic agents [73]. Therefore, the presence of a repair focus is not an ultimate piece of evidence demonstrating the existence of a DSB at a given site in the nucleus at the time of observation.

Finally, it is also important to note that methods that belong to a class of indirect genomic approaches exploit cellular DNA repair mechanisms; therefore, they detect DNA repair events rather than the damage itself. When error-prone repair (NHEJ) occurs, a DNA break that activated DDR should become detectable, but when an error-free process (HR) is involved, a DSB will not be detected.

## Capabilities of selected genomic methods

### DSB mapping utilizing massively parallel sequencing (DSB sequencing)

Genomic methods have revolutionized the study of DSBs in the last decade, allowing genome-wide mapping with single-nucleotide resolution, beginning with BLESS [12] in 2013. DSBs can be mapped by sequencing either through direct break detection (Fig. 3, top left) or indirectly, by detecting proteins involved in DDR or specific DSB repair products (Fig. 3, bottom left). As discussed earlier, the capabilities of various genomic techniques differ substantially.

The direct sensitivity of genomic methods is rarely assessed by mixing known proportions of cells containing a DSB induced at a specific locus with intact control cells. However, for the two techniques where such validation was performed, the results were remarkable: a detection threshold of 1 in 10 000 cells for END-seq and 1 in 100 000 cells for i-BLESS was reached. More traditionally, the sensitivity of DSB detection methods has been evaluated by the number of cutting sites of a given restriction enzyme (e.g. AsiSI) that can be successfully identified. Defined this way, sensitivity has steadily increased over the years. However, to keep these results in context, it is important to remember that along with genuine major improvements in methodology, these tests show increasingly better results simply due to parallel advancements in sequencing throughput. Moreover, different methods are often tested in ways that are not directly comparable, using different restriction enzymes to determine the percentage of captured cut sites or different experimental designs to estimate synthetic ‘1 DSB in N cells’ metrics, as was the case for END-seq and i-BLESS.

Sensitivity, simplicity, bias, and capacity to quantify DSBs by various genomic methods, discussed here and above, are summarized in Table 3. The DSB quantification capabilities of the genomic methods will be discussed below.

**Table 3.** Sensitivity, simplicity, bias, and capacity to quantify DSBs by various genomic methods (Y -Yes, N - No)

Method	Sensitive	Simple	Unbiased	Quantitative
BLESS	N	N	N	N
i-BLESS	Y	N	N	N
END-Seq	Y	N	N	N
BLISS/sBLISS	Y	Y*	N	N
qDSB-Seq (labeled as in BLESS)	N	N	N	Y
qDSB-Seq (labeled as in i-BLESS)	Y	N	N	Y
INDUCE-Seq	Y	#	Y	N

* in later versions of the protocol,

# - proprietary method, details not publicly available.

### Indirect genomic methods of DSB detection

An example of a genomic indirect DSB detection method is chromatin immunoprecipitation followed by sequencing (ChIP-seq) against a DDR protein, e.g. γH2A.X to detect DSBs, or XRCC4 or RAD51 to detect a specific type of repair (NHEJ versus HR). ChIP-seq-based methods are currently not used as a primary tool for DSB detection due to low resolution and bias, but they are still used to classify DSBs [77]. Other indirect methods use DSB repair products and a ‘bait’ oligo with a known sequence to identify DSBs. For example, DSB bait and the unfaithful NHEJ DNA repair pathway (IDLV, GUIDE-seq) or translocations (TC-Seq [28], HTGTS [52], LAM-HTGTS [29]) are exploited to capture DSBs (Fig. 3, bottom left). Consequently, such methods detect only DSBs unfaithfully repaired by NHEJ or exclusively in translocation-prone regions. Additional problems may arise due to the varying efficiencies of DNA repair and transfection between cells and the potential toxicity of the latter. Moreover, these methods [28, 29, 52] detect DSBs accumulated over several hours (transfection time) and, unlike direct methods, do not provide a precise temporal snapshot.

### Quantification of DSBs using genomic methods

Most genomic methods do not provide information on the absolute number of DSBs in individual cells or the average number of DSBs per cell within a population. Instead, these approaches measure the relative frequencies of encountering a DSB at specific genomic loci. This distinction has led to persistent semantic confusion in the literature. For example, [46] reported that the DSBCapture method identified 84 946 DSBs in a normal human epidermal keratinocyte cell line, meaning that DSBs were detected at 84 946 distinct genomic coordinates, rather than representing either the average number of DSBs per cell or the total sum of physical breaks in the sample.

Because absolute DSB counts per cell are of great interest and necessary to evaluate the physiological impact of DSBs, several attempts have been made to design a genomic method to simultaneously map and quantify DSBs on a per-cell basis.

These methods fall into two categories. The first approach to quantifying DSBs is to simply count them accurately in the sample and divide by the number of cells used, thus creating a reasonable estimate of the average number of DSBs per cell in a sample. Some genomic methods, most notably BLISS and INDUCE-seq, were initially proposed as viable strategies for quantification of DSBs using this approach. Both techniques, either by utilizing UMIs or by bypassing PCR entirely, possess the theoretical capability to count sequenced DSBs directly. In principle, every INDUCE-seq read—and every BLISS read cluster with a unique UMI—corresponds to an individual DSB. Consequently, one might assume that, by knowing the total number of sequenced DSBs and the number of starting cells, the average number of DSBs per cell could easily be calculated. However, the large-scale loss of material inherent to the DSB labelling and sequencing protocols renders these estimations highly inaccurate (Fig. 5).

**Figure 5.**
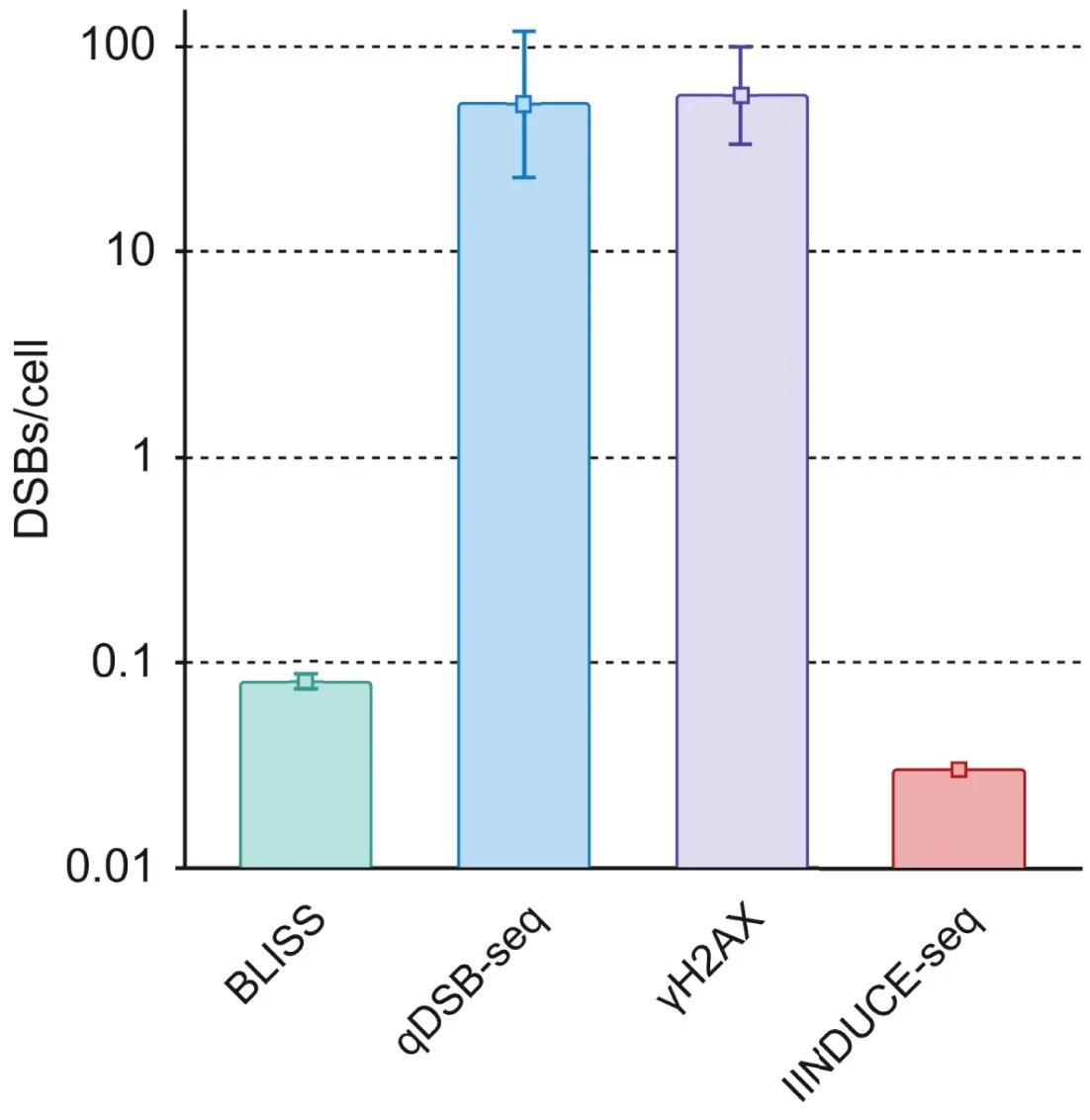
Comparison of the quantification of AsiSI-induced DSBs in DivA cells using BLISS, qDSB-Seq, INDUCE-Seq, and counts of γH2AX foci. AsiSI-induced DSBs in cells were independently quantified using three genomic methods—BLISS [47], qDSB-Seq with BLESS labelling [49], and INDUCE-Seq [51]—and by immunofluorescence detection, by two independent studies counting γH2AX foci in individual cells [78, 79]. DSBs per cell counts for the BLISS, qDSB-Seq, and γH2AX studies were calculated as explained in detail in [49]. For INDUCE_Seq, the average DSBs per cell were calculated by dividing the total number of reads mapped to AsiSI sites by the number of cells used, based on the data reported by Dobbs [80]. Due to the quantification results of the BLISS and INDUCE-Seq methods that vary by several orders of magnitude from qDSB-Seq and γH2AX-based quantification, the logarithmic scale on the *y*-axis was used to allow presenting all results in one figure (Created in BioRender. Dobrucki, J. (2027) https://BioRender.com/ed5cawe).

Another approach to DSB quantification is represented by qDSB-Seq (Quantitative DSB sequencing), the only technique specifically designed to not only map but quantify DSBs in terms of their absolute counts per cell. qDSB-Seq is using spike-in DSBs for calibration. Such calibration DSBs are induced by a site-specific restriction endonuclease (either *in vitro* or *in vivo* via engineered expression) at predetermined genomic loci. Then, in addition to regular DSB sequencing, genomic DNA sequencing (or qPCR) is performed at specific restriction enzyme recognition sites to determine the exact cutting efficiency, which is later used for calibration. qDSB-Seq was extensively tested to show that the quantification it offers is stable, independent of the specific restriction enzyme chosen and the type of DNA ends induced, and that it works well even with a single spike-*in site* per genome while yielding results consistent with image-based quantification of DSBs (Fig. 5) [49, 50]. A crucial advantage of qDSB-Seq is its reliance on internal control, which ensures that the method is not prone to sequencing artifacts.

Thus, at least at the current stage of technology development, only spike-in-based quantification techniques (such as qDSB-Seq)—where calibration DSBs are exposed to the exactly the same processing and material loss as the DSBs have the capacity to deliver truly accurate DSB quantification.

## Capabilities of selected imaging methods

The sensitivity, specificity, and capacity to quantify DSBs by selected imaging methods vary substantially and are briefly discussed below.

### Sensitivity

To be useful in various studies of DNA damage, an imaging method for detecting DSBs should be able to detect a wide range of DSB numbers, starting from one individual lesion in a cell nucleus. However, this requirement is difficult to fulfil due to various limitations of the existing methods.

Indirect imaging methods for detecting DSBs vary widely in their sensitivity. Recruitment of the repair protein 53BP1 and phosphorylation of histone H2AX result in many protein molecules concentrated near the DNA lesion; thus, their detection by immunofluorescence constitutes a sensitive quantification approach (provided that a typical immunofluorescence background is low). On the contrary, detection of DSBs by imaging foci of repair proteins, which are recruited in small numbers in relation to the unbound/mobile pool (such as Ku70/80), is expected to be less sensitive. Moreover, it is often difficult to know whether a repair focus detected by microscopy represents one DNA lesion or a cluster of DSBs. The CRISPR/Ca9 system offers a rare opportunity to induce and subsequently detect a known number of breaks in a cell [19].

A direct DSB detection method, the TUNEL assay, relies on immunofluorescence detection of nucleotide analogues incorporated at a broken DNA end. The number of these nucleotides added at one DSB is unknown and apparently not sufficient to detect individual lesions. Immunofluorescence is associated with a detectable signal background arising from residual nonspecific binding of antibodies. The signal-to-noise ratio of such images is poor, and weak signals remain undetected. As a result, the TUNEL assay is a low-sensitivity approach in which DNA breaks are represented by a general level of fluorescence in the cell nucleus or apoptotic bodies, rather than individual foci [81]. It is used for the detection of apoptosis by detecting DNA breaks, which occur in very large numbers when DNA is degraded by caspase-activated DNase. However, detection of individual DNA breaks is not possible.

The comet assay relies on pulling DNA fibres out of the nucleus of a fixed cell after DNA breakage. The nature of the assay makes it indirect and imposes the need for calibration. The sensitivity of this approach is believed to be sufficient to detect 100 or more DSBs [82] but not individual lesions or low numbers of DSBs; therefore, precise studies of endogenous DSBs are not feasible. The original subnuclear position of DSBs remains unknown.

The highest sensitivity among existing methods is offered by dSTRIDE, which was shown to be capable of detecting individual DSBs induced in a predetermined genomic locus by CRIPSR/Cas9 system [19]. dSTRIDE also successfully detected DSBs in untreated human fibroblasts, sperm, and other cells. Although the ability to detect individual lesions has been proven, so far no rigorous study of the yield of the detection of individual breaks by dSTRIDE has been published.

### Specificity

The specificity of detection of DSBs by microscopy is a key in any attempt to quantify DNA damage. Although histone H2AX phosphorylation is generally considered a marker of DSB, assays based on the detection of γH2AX are expected to have limited specificity, as weak phosphorylation of histone H2AX may occur in the absence of DSBs [73]. The specificity of γH2AX as a marker of DSBs is also questioned by the observation of strong signals of immunodetected γH2AX in oocytes in chromosomal regions without apparent discontinuity [83]. Using 53BP1 as a marker of DSB is limited by the fact that this protein is involved only in NHEJ [84].

The specificity of dSTRIDE hinges upon the ability of the TdT enzyme to add mononucleotide analogues exclusively to DNA ends at DSBs but not at SSBs. Although this specificity has been questioned, we are not aware of any systematic study on the subject. Although TdT was discovered over 60 years ago, new information about its catalytic properties emerged much later. It was shown that TdT can exhibit an *in trans* template-dependent activity [85]. It is unclear whether this activity can in any way influence the specificity of DSB detection methods that employ TdT. The specificity of dSTRIDE could also be influenced by the antibodies used in this assay. In dSTRIDE, fluorescence signals arise only when two different antibodies are bound to adjacent nucleotide analogues marking a DSB. In contrast to typical immunofluorescence, in dSTRIDE, sparse nonspecific binding of secondary antibodies does not result in any fluorescence signal, as long as the specificity of primary antibodies that recognize mononucleotide analogues is high. However, nonspecific binding of primary antibodies can result in false-positive signals and deprive PLA of its prime advantage [86]. In general, the specificity of dSTRIDE in detecting DSBs is expected to be very high.

### Quantitation

Quantifying DSBs by imaging relies on detecting individual DNA repair foci formed by recruited repair factors (MRN complex, ATM, 53BP1, BRCA1, etc.) or the foci of post-translationally modified histones (γH2AX). In the first approximation, the number of these foci represents the number of DSBs. These assessments may, however, represent under- or overestimates of the actual number of DSBs, as discussed below. We now focus on the technical aspects of popular DSB detection based on histone H2AX phosphorylation, recruitment of 53BP1 or other repair factors, and direct detection of DNA ends by dSTRIDE.

#### Detection of DSBs based on γH2AX foci

Potential reasons for underestimating the number of DSBs include a typically large size of γH2AX foci. It is impossible to establish the true number of DSBs embraced by one such focus. Some correlation between the size of the focus and the number of DSBs has been shown [87]; however, proper calibration of the focus size to count the number of DSBs embraced by this focus does not appear to be possible. As a result, multiple clustered DSBs cannot be distinguished from individual breaks.

Another potential reason for underestimating the number of DSBs might be the local movement of damaged chromatin and the coalescing of DSBs into one local focus of active DNA repair. This process was observed in DivA cells [79] and could be a source of underestimating the number of lesions.

It is important to note that not only when the repair foci marking DSBs are relatively large (as for γH2AX or 53BP1) but also when they have a small diameter (e.g. Rad51 foci), limited spatial resolution of the fluorescence images is still expected to result in foci overlapping and some underestimation of the number of DSBs. In the case of numerous breaks, this problem may, but only to some extent, be alleviated by using super-resolution imaging [88].

The limited specificity of the antibodies used in the immunofluorescence detection of repair foci results in detectable noise levels. This problem is expected to result in an underestimating the number of DSBs, as the signals of weakly stained foci may become undetectable above the level of nonspecific signals that are typically present in immunofluorescence images.

Although the technical issues listed above can lead to an underestimation of the number of DSBs in a cell nucleus, at least one factor can lead to an overestimate. As mentioned earlier, there is some evidence suggesting that some γH2AX foci may be unrelated to DNA breaks [73, 74, 89], which could lead to false-positive results.

#### Quantification of DSBs based on recruited repair factors (e.g. ATM, 53BP1, Ku, and others)

Some DNA repair factors are recruited in very high numbers of copies and remain at the damage for a long time; therefore, they are detected by microscopy readily (e.g. 53BP1). Other proteins are recruited in smaller numbers of copies and remain at the damage site for a short time (e.g. Ku70/80, ligases); thus, their detection is technically more difficult. Possible detachment of proteins from their targets during a fixation process can be an additional complication in indirect DSB detection methods [90, 91].

The detection of repair factors recruited to damage by microscopy is complicated by the fact that these proteins are also present throughout the nucleoplasm. Therefore, the local concentration of a repair factor in a repair focus must be significantly higher than the concentration in the nucleoplasm to allow detection. Considering that the concentration of most repair factors is in the range of thousands to tenths of thousands [92], it becomes clear that recruitment of 10 or even 100 molecules to a DNA lesion is undetectable in the presence of a large nucleoplasmic pool of this factor. This also implies that damage-recruited proteins can remain undetected not because of poor sensitivity of the fluorescence detector but because of the presence of a mobile/unbound pool of this protein in the nucleus.

There are several quantitative studies on the concentration of repair factors in the whole nucleus and within nuclear foci [92–95] and the concentration of all proteins in repair foci [96]. The total number of 53BP1 molecules in the U2OS nuclei was estimated as 2.4 × 10^5^ by [94] and 1.2 × 10^4^ (measured by Halo tagging and western blots) by [92]. Interestingly, a report [93] demonstrates that 1200 53BP1 molecules are present in a repair focus (293FT human kidney cells, damage induced by ionizing radiation), while [94] reveals that 4.6 × 10^3^ 53BP1 molecules are found in individual DSB repair foci (when damage was induced by a radiomimetic drug) in U2OS cells. This means that 53BP1 molecules recruited to damage may be in the range of 1%–10% of the total amount of this protein. Interestingly, considering that an average protein concentration in a cell is ~2 – 4 × 10^6^/µm^3^ [97], even if all copies of 53BP1 were recruited to a damaged site, they would make up <0.1% of all proteins already present there.

These considerations hint at potential technical difficulties in detecting DSB repair foci by live-cell imaging or immunofluorescence. They also raise the question of whether the presence of detectable repair foci signals the presence of one DNA lesion or a cluster. It is possible that some individual lesions are not accompanied by a detectable number of repair factors, especially when the number of DSBs is high, such as after exposure to genotoxic treatments. One has to consider that in live cells expressing fusion proteins, only some of the recruited repair factors are fluorescently tagged since endogenous proteins are recruited, possibly even more efficiently. In addition, when immunofluorescence is considered, some but not all of the recruited molecules of repair factors are bound by an antibody.

#### Detection of DSB by imaging foci of 53BP1

Immunofluorescence detection of 53BP1 foci recruited to damage poses problems similar to those described for γH2AX, that is, the large size and overlap of such foci. On the other hand, 53BP1 tagged with FP offers the possibility of studying dynamic processes and observing the accumulation of this protein in the region surrounding the DNA lesion, and a decrease in local concentration of 53BP1 after completion of repair. However, foci containing a low number of 53BP1-FP molecules may be undetectable due to the evenly distributed fluorescence of the mobile pool of this protein. 53BP1 foci are relatively large; therefore, conclusions about the number of DSBs that gave rise to a repair focus cannot be drawn. Additional uncertainty (and a possible overestimate) comes from the fact that some 53BP1 foci may be unrelated to DSBs [86]. Another complication comes from the fact that the recruitment of 53BP1 to DSB is cell-cycle dependent [70, 71] and that the recruited protein can form large repair foci of complex architecture.

#### Quantitation of DSBs based on dSTRIDE

Although dSTRIDE stands out as the only method capable of detecting individual DSBs directly, assessing the true number of DSBs may still pose some problems. Since the procedure involves the use of primary and secondary antibodies, their unimpeded access to broken DNA ends is critical. Dense cross-linking by formaldehyde during cell fixation and a high density of heterochromatin may limit the access of antibodies and result in underestimating the number of DSBs. Other factors include the need to detect DNA ends other than 3′, and uncertainty about the effectiveness of cleaning ‘dirty’ ends by enzyme methods, if used. The phenomenon of repair foci coalescing can influence the final count of DSBs. Finally, the relatively large size (~200 nm in diameter) of a long concatemeric DNA, a product of the RCA reaction, makes it impossible to count all DSBs detected by dSTRIDE if the number of lesions reaches hundreds, as would be the case in cells exposed to genotoxic agents.

The sensitivity, specificity, capacity to quantify DSB, and complexity of several imaging methods are summarized in Table 4.

**Table 4.** Sensitivity (an ability to detect individual DSBs), specificity (an ability to minimize signals unrelated to DSBs), quantitation (an ability to measure the absolute number of DSBs in a cell), and assay complexity (the degree of complexity of the protocol, the expertise required to perform it, and the time needed) of various imaging methods of detecting DSBs (+ low, ++ moderate, +++ high)

Method	Sensitivity	Specificity	Quantitation	Complexity
dSTRIDE	+++	+++	+++	+++
TUNEL	+	++	+	+
Comet assay	+	+	+	++
Recruitment of repair factors	+++	++	++	++
Phosphorylation of histone H2AX	+++	++	++	+

## Discussion

Various early studies in the field of radiobiology and other areas suggested that the number of endogenous DSBs in mammalian somatic cells and oocytes ranges from zero to just a few dozen [98–100]. It was tacitly accepted that larger numbers of DSBs present simultaneously in the nuclear DNA of a cell result in cell death. Single-cell imaging methods yield a relatively consistent assessment of the number of endogenous DSBs found in cells in tissues and in *in vitro* cultures. These numbers, primarily based on the detection of γH2AX or 53BP1 foci, range between 0 and 50 or so per nucleus at any given time, confirming earlier studies [74].

Genomic methods revolutionized the study of DNA DSBs by providing accessible protocols to map DSB frequencies genome-wide at single-nucleotide resolution. However, studies employing these genomic approaches frequently report DSB frequencies that differ by up to three orders of magnitude from values inferred via imaging. Interestingly, these genomic estimates swing drastically in both directions. For example, some protocols report tens of thousands of DSBs in cell nuclei [46, 80]. In contrast, other genomic studies yield estimates three orders of magnitude lower than imaging data [49]. Specifically, in the well-characterized DivA system, standard AsiSI induction was shown to produce 57 ± 33 DSBs per cell based on γH2AX foci counting by Caron *et al*. [79] and Iannelli *et al*. [78]. However, when two independent groups used two genomic methods, BLISS and INDUCE-seq, to estimate these same AsiSI-induced breaks [47, 80], their results indicated that, on average, less than one DSB was induced per cell, strongly contradicting prior literature [99]. Crucially, qDSB-Seq eliminates these discrepancies; this dedicated quantification protocol produces 52 ± 65 AsiSI-induced DSBs in DivA cells, restoring consistency with established imaging studies.

In principle, when such severe discrepancies arise between two classes of methods, imaging-based methods, genomic methods, or both could be severely in error. The systematic analysis of the principles underlying these two groups of methods provided in this review explains the root of this discrepancy. In imaging, DSB quantification is straightforward because it relies on the direct observation of cellular responses or chemical reactions. On the contrary, accurate quantification of DSBs using sequencing requires a dedicated approach like [49], which most genomic methods do not yet employ but which can easily be incorporated into a genomic method. Currently, the core strength of genomic approaches remains the unprecedented, near single-nucleotide resolution with which they map DSBs, while imaging approaches excel at providing accurate counts of DSBs in individual cells but offer little information regarding their genomic coordinates.

Unlike in imaging methods, in a typical genomic method the estimate of total DSBs is not obtained directly, since, due to amplification and loss of material during processing, the number of sequencing reads detected is not an obvious function of the number of DSBs in the sample. Two methods, BLISS and INDUCE-seq, solved the problem of obtaining multiple sequencing reads from a single labelled DSB end. In INDUCE-seq, PCR amplification is simply eliminated; in BLISS it is still used, but thanks to UMIs, PCR duplicates can be removed during data analysis. These innovations ensure that there would be no more DSB-originating reads detected then original DSBs in the sample. In other words, the total number of DSBs in the sample will not be overestimated by counting DSB-originating reads (in BLISS, including only one read per each unique UMI). However, underestimating the number of DSBs in the sample is still an issue. To avoid underestimation in read counting methods, no loss of material would have to occur during DSB labelling and sequencing, which is, intuitively, very far from true. Indeed, DSB labelling is a complex, multistep, multi-day process, where loss of material can occur at every step. Even standard Illumina sequencing protocol includes sequencing only 12.5% of the sample after amplification. All these losses of material at different steps eventually lead to the sequencing of <<1% of the DSBs present in the sample, according to our estimates presented in Fig. 5.

Therefore, a seemingly intuitive method of counting the cells in the sample using the cell counter and later dividing by this value the number of sequencing reads corresponding to individual DSBs does not work. As Fig. 5 shows, methods (BLISS & INDUCE-seq) attempting to quantify DSB will results in orders of magnitude underestimation. Such approaches will be able to estimate DSBs per cell accurately only once—or if—we will reach near-perfect retention of starting material, which is far from the current technology development. Alternatively, spike-in DSBs can be used, on top of existing protocol.

In contrast, the spike-in approach to quantifying DSBs per cell (qDSB-Seq) does not require low material loss during processing, since spike-in DSBs will be lost in such scenario at roughly the same proportion as DSBs of interests, so quantification will not be negatively impacted.

Although imaging methods may provide underestimates of DSBs (e.g. for DSBs close in space, which may be resolved as a single focus), as described in this review, the fact that approaches based on direct detection of DNA breaks as well as methods employing DDR markers yield consistent results supports the notion that these assessments—generally in the range of 0 to 50—are trustworthy.

It is also interesting to consider why some genomic methods that attempt to quantify DSBs provide results that vary by up to three orders of magnitude from imaging-based estimates. There are a couple possible explanations for these discrepancies.

First, when DSBs are distributed highly unevenly throughout a population, where a small fraction of cells carry the vast majority of damage, genomic methods are at an inherent disadvantage [101]. Because sequencing naturally measures a population average, the resulting value will vary wildly from the median; in contrast, single-cell imaging prevents the true distribution from being completely obscured by outliers. However, in the study presented in Fig. 5, this challenge was not present since we were only analyzing DSBs induced at AsiSI cutting sites. Second, some genomic methods apply quantification approaches (e.g. BLISS, INDUCE-seq) that are seemingly straightforward and intuitive but ultimately fail in practice (Fig. 5) due to underlying technical limitations, discussed earlier.

### Averaging genomic DSB data over a heterogeneous population—the role of apoptotic cells

Currently, DSB frequencies can be accurately estimated genome-wide in pooled, but not individual, cells. The numbers of endogenous DSBs per cell vary widely, as observed in many microscopic studies before, and recently, heterogeneity in cell population within a standardized system such as DIvA was observed [102], mediated by cell cycle and chromatin context, resulting in differing repair factors recruitment. Such heterogeneity of cellular environment as a cause of observed heterogeneity of DSB repair was postulated, among others, in [101], where subpopulations of cells were analysed computationally with reference to their differing DSB repair.

Untreated human cells generally exhibit 0–6 DSBs (based on γH2AX foci) [73, 103], while apoptotic cells (with DNA digested into units of nucleosome size) can carry millions. Consequently, a small subpopulation of cells with many DSBs can eclipse DSB measurements from the majority of cells. *In vitro* cell cultures typically contain some 2%–5% apoptotic cells [104]. Let us consider an example in which DNA is collected for DSB sequencing. About 3% of these cells are apoptotic. The estimated total number of DNA fragments in a diploid apoptotic human cell can reach ~3 × 10^7^, representing as many breaks (and 12 × 10^7^ DNA ends). If one considers DSBs in a population of 10^3^ cells containing 3% apoptotic cells, the number of DNA breaks in all 970 non-apoptotic cells could be 1 × 10^4^, while the number of DSBs in the 30 apoptotic cells could be 9 × 10^8^. Thus, the average number of DSBs present in all the DNA extracted from this whole cell population will be at least ~9 × 10^5^ per cell, i.e. even five orders of magnitude higher than the average number of DSBs in the non-apoptotic cells. This simple example shows that even a small proportion of apoptotic cells with degraded DNA can seriously distort the assessment of the number of DSBs by most currently used sequence-based methods. Even if 99% of the apoptotic cells were removed from the analysis, the result would still be in the order of 1 × 10^4^ DSB/cell, a strong misrepresentation of the number of breaks in non-apoptotic cells, and a result which is highly inconsistent with the imaging data.

This challenge can be circumvented by a careful analysis, but advance methods specifically designed to analyse DSB distribution in distinct sub-populations need to be used. Moreover, some additional data (e.g. FACS data, and/or pulse-field gel electrophoresis, or some imagining data [101]) are needed in addition to DSB sequencing to construct a model of these subpopulations. Also, techniques for studying DNA damage and repair are being developed, which provide more data for studying heterogeneity of DNA damage and repair [102].

### Classes of DNA breaks that are challenging for genomic and imaging methods

Imaging methods cannot distinguish clusters of lesions from individual breaks due to the physical size of fluorescent labels [thousands of γH2AX histones, molecules of FP-53BP1, or other recruited repair factors, or DNA concatemers (products of the RCA reaction) in dSTRIDE] and limited spatial resolution of the images, as described in the previous sections.

Genomic methods excel in detecting and interpreting data in which DSBs group or cluster in certain genomic regions. However, experiments that yield fairly constant DSB frequencies across the genome (e.g. DSB patterns resulting from exposure to ionizing radiation) are very difficult to interpret without information about absolute DSB counts (i.e. average DSB per cell numbers) [49]. This information can be obtained by inducing DSBs in predetermined positions that are later used in calibration, as in qDSB-Seq [49]. Knowing absolute DSB counts allows for between-sample comparisons, i.e. assessing if the average level of DNA damage in the sample is going up or down. However, because DSB sequencing methods inherently measure averages, even with the added benefit of knowing the average number of DSBs per cell, interpretation can be challenging: without a lot of extra effort and data [49], it is impossible to conjecture how those values change in most cells, since DNA damage levels tend to be so high in a small minority of apoptotic cells that they can easily overshadow trends occurring in the broader population. Here, imaging methods shine, as median population values are easily inferred from such data. Similarly, it is difficult to interpret what ‘background’ is in sequencing methods. In earlier methods, a substantial part of a background was due to artifacts induced by overfixation and inadequate protein removal (Fig. 6). However, even the current, much lower background level, when used to calculate average DSB numbers per cell, leads to results orders of magnitude higher than the known maximal tolerable DSB burden for cells [105]. Moreover, those inferred average DSB counts per cell are also orders of magnitude higher than those measured by imaging methods. Therefore, it is clear that those values are far from median values, but it is currently not clear which percentage of detected background DSBs originate from endogenous breaks or apoptotic cells, and how many of them are artifacts of either labelling or sequencing. We hypothesize that when modern DSB labelling and Illumina sequencing methods are used, most of those background breaks originate from a minority of highly damaged cells in the population, but more research is needed to rigorously verify this hypothesis. DSB sequencing methods are also not appropriate for estimating median (i.e. typical) levels of spontaneous DSBs in cells, unless such breaks occur in specific genomic positions (e.g. Spo11p-catalyzed DSBs in meiosis [106]).

**Figure 6.**
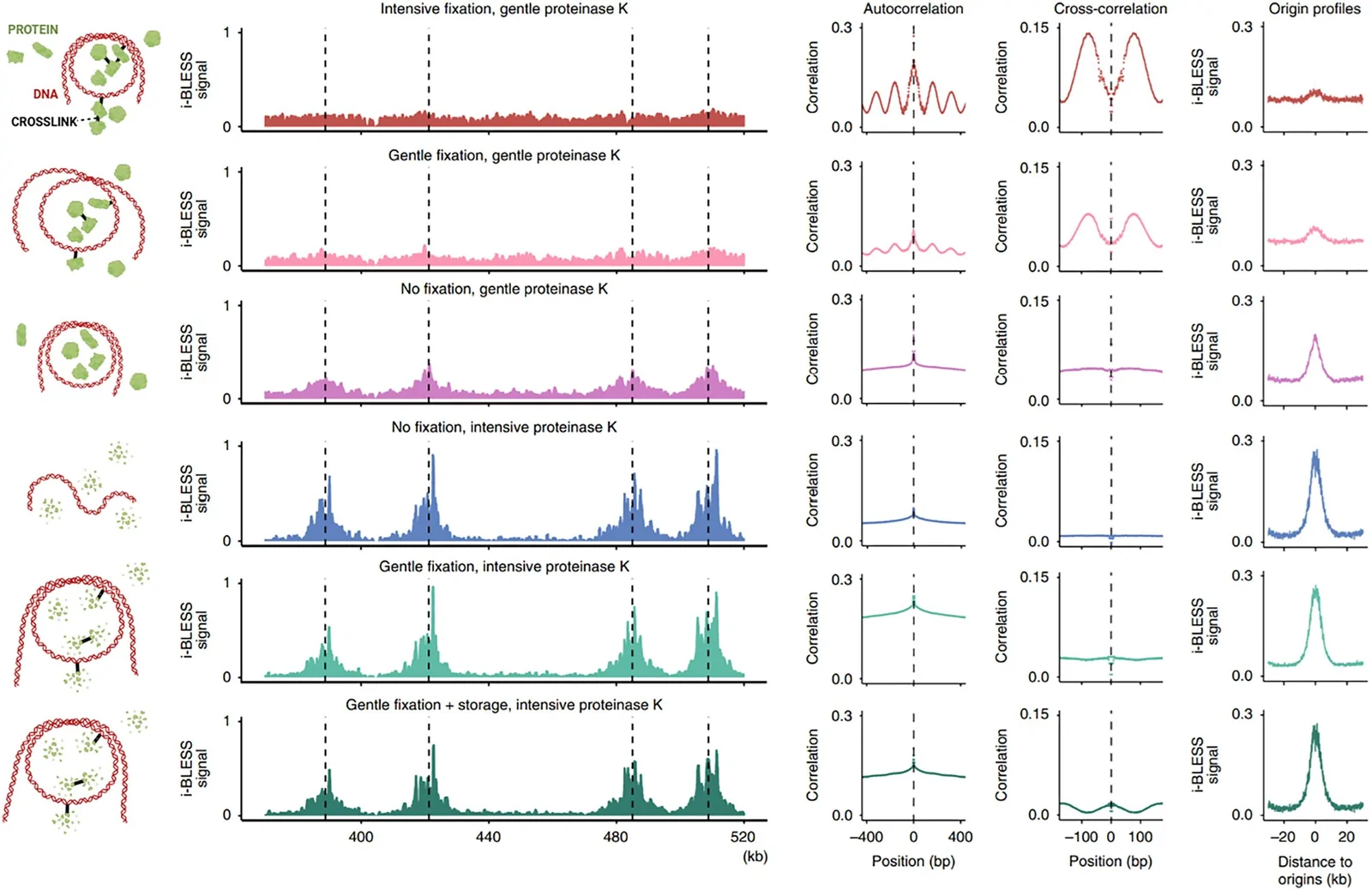
Impact of the parameters of the experimental protocol (affecting DNA accessibility) on the quality of DSB sequencing data using the example of i-BLESS [40, 48]. Protein degradation and the resulting changes in chromatin structure are shown symbolically in the left column. *mec1-1 sml1-1* yeast cells were treated with HU and subjected to the indicated treatments: intensive fixation: cell fixation with 2% formaldehyde for 30 min; gentle fixation: cell fixation with 2% formaldehyde for 5 min; storage: storage of fixed cells for 7 days at 4°C; intensive proteinase K: 50 µg/ml overnight at 50°C; and gentle proteinase K: 1 µg/ml for 5 min at 37°C. For each sample, the i-BLESS signal around replication origins (dotted vertical lines) in a representative region of chromosome VII, the autocorrelation of the i-BLESS signal, the cross-correlation of the i-BLESS data with MNase-seq data [107], and averaged i-BLESS signal around replication origins are shown. i-BLESS data in the top two panels, for which signal-to-noise ratio is the lowest (as illustrated by averaged meta-profiles of i-BLESS signal around replication origins), shows clear periodicity in autocorrelation pattern related to nucleosome spacing, suggesting that is the main source of noise during DSB detection. The i-BLESS signal intensity was calculated by normalizing read count to 1 million reads total (Created in BioRender. Dobrucki, J. (2027) https://BioRender.com/0xmdlct).

As mentioned earlier, to interpret the DSB sequencing data, it is important to know absolute (i.e. per cell) DSB counts. Therefore, several methods were created to allow quantitative sequencing of DSBs. Two methods (BLISS and INDUCE-Seq [47, 51]) base their estimations on calculating the numbers of unique DSBs detected in the sample and dividing it by the number of cells used in the experiment. During library preparation, individual DNA fragments are amplified by PCR, thus creating multiple identical fragments, resulting in the possibility of an individual DSB being sequenced multiple times, making it difficult to assess the total number of unique DSBs. BLISS and INDUCE-Seq solve this problem differently. BLISS utilizes unique molecule identifiers (UMIs) to label individual breaks. Due to the uniqueness of UMIs, despite PCR amplification, multiple reads originating from the same DSB can be combined during data analysis. INDUCE-Seq leveraged Illumina sequencing, which now requires much less starting material, and simply removed read amplification via PCR from the protocol, thus preventing creating multiple reads originating from individual DSBs. Both approaches successfully solve the problem of potentially counting individual DSBs as multiples; however, neither solves the problem of missing DSBs during labelling, library preparation, sequencing, or data analysis.

In fact, according to Illumina specifications, during the sequencing step, only 12.5% of the staring material is loaded on the sequencer. Furthermore, the sequencing load efficiencies vary between ~25% for NextSeq and only 0.03%–0.5% for MiSeq. Thus, the sequencing step alone results in a loss of at least 97% and up to 99.96%. Furthermore, according to our calculations (Fig. 5), the total material loss in the published BLISS and INDUCE-Seq data from DivA cells is 700-fold and 2000-fold, respectively, allowing us to roughly estimate that 95%–98% of the starting material is lost during the DSB labelling procedure. This is not surprising considering the complexity of many steps involved in DSB labelling procedures. It also does not mean that genomic methods cannot be used to successfully quantify average DSB numbers in the population in cases where such numbers are not highly variable between cells. The example of qDSB-Seq shows that procedures employing empirical calibration—in the case of qDSB-Seq spike-in DSBs—can still successfully estimate the true average numbers of DSBs in the samples, since spike-in DSB counts will be affected by material loss in the same ways as counts of DSBs of interest, and their absolute frequency is estimated by methods immune to material loss (e.g. qPCR or genomic DNA sequencing; for a detailed explanation see [49]). However, ignoring this loss of material in DSB quantification, as is implicitly done in [47, 51] leads to inferred DSB numbers being three orders of magnitude off (Fig. 5).

### The language describing the results of genomic methods

Another source of confusion is that in some papers a list of DSBs is constructed and described as a ‘number of DSBs’ detected in a given cell type. This is a rather misleading phrasing, since this is a list of all detected DSBs, not DSBs detected in a typical cell; DSB sequencing methods do not have the capability to detect the latter without additional data and advanced analysis [101]. Furthermore, with the increased depth of sequencing, DSBs in all mappable positions would likely eventually be detected in all genomic locations, so such a ‘DSB number’ is NOT a meaningful characteristic of a given cell type. Unlike, average DSB/cell count, a number of DBSs detected in a given cell line is not very informative. Moreover, such a numer is not particularly meaningful. We recommend instead to number, since in addition to a typical number of DSBs in a given cell type and their genomic distribution, it will be heavily skewed by the chosen sequencing depth, sensitivity, and efficiency of the DSB labelling method, and the criteria for identifying DSBs.

## Conclusion

Imaging and genomic methods are inherently different and have unique advantages and disadvantages. Therefore, it is not surprising that they not only yield different types of information, namely the number of DSBs per nucleus versus the average probability of DSB induction in a given genomic position, but also often give extraordinarily different assessments of the expected numbers of endogenous DSBs in cells.

Genomic DSB detection methods have matured substantially since 2013, when BLESS was first introduced. The newer methods now exhibit much lower background levels (Fig. 6) that facilitate data interpretation without the need to rely on more advanced data analysis techniques [48].

Some of the DSB techniques were developed intentionally with clinical use in mind, e.g. the very low starting material requirement in BLISS was making it more feasible for using for patient samples, including cancer samples previously preserved on glass slides. DSB sequencing methods proved also key in studying and monitoring off-target DSBs. During gene editing (e.g. CRISPR-Cas9 [108], ZFN [109], TALENs [110]), nucleases induce programmed DSBs. Since nucleases are not 100% accurate, off-target DSBs are also created [111–113]. Therapeutic gene editing is done in a large number of cells; therefore, even rare off-target DSBs pose significant health risks. Indeed, some trials of gene-editing therapies were halted due to patients developing chromosomal abnormalities, new disorders, or even dying [112–114]. There is now consensus [115–117] that accurate detection and quantification of off-target DSBs, using multiple methods [117], is key in developing safe gene therapies. There is also continuous effort to improve fidelity of gene-editing therapies, i.e. to eliminate off-target editing [118].

Interestingly, as the various DSB detection techniques are improved, the results of DSB measurements by imaging and genomic methods appear to be converging (except for some cases described earlier) and reflect the true numbers of DSBs more accurately. Imaging methods have been improved from a situation where only a general level of DNA damage could be assessed (as in TUNEL assay or comet assay) and the specificity of detection was limited, to a level where specific and highly sensitive detection of individual DSBs (dSTRIDE) is possible, and the number of detected breaks can reasonably be expected to reflect reality well. On the other hand, the development of dedicated quantification approaches caused genomic methods to provide estimates of DSB counts more consistent with those observed by microscopy, except in the inherently most challenging case for genomic methods, when DSB counts vary orders of magnitude between cells. One should expect that further improvements in both groups of techniques will eventually lead to even more convergence and highly accurate measurements. However, the fundamental differences in genomic and imaging methods we discussed here in detail, i.e. genomic methods providing single nucleotide positional resolution but population-averaged DSB frequencies, while imaging methods much better estimating true DSB counts in individual cells but lacking sufficient spatial resolution, will still remain true. Thus, there will always be huge benefit to analysing and integrating these data tighter to obtain comprehensive, multiscale, and multicell view of DNA damage and repair processes.

## Data Availability

This is a review paper; it does not contain new experimental data.

## References

[1] Boveris A, Cadenas E. Mitochondrial production of superoxide anions and its relationship to the antimycin insensitive respiration. FEBS Lett. 1975;54:311–4. 10.1016/0014-5793(75)80928-8 236930

[2] Epe B, Pflaum M, Häring M et al. Use of repair endonucleases to characterize DNA damage induced by reactive oxygen species in cellular and cell-free systems. Toxicol Lett. 1993;67:57–72. 10.1016/0378-4274(93)90046-Z 8383892

[3] Lindahl T . Instability and decay of the primary structure of DNA. Nature. 1993;362:709–15. 10.1038/362709a0 8469282

[4] Mehta A, Haber JE. Sources of DNA double-strand breaks and models of recombinational DNA repair. Cold Spring Harb Perspect Biol. 2014;6:a016428. 10.1101/cshperspect.a016428 PMC4142968 25104768

[5] Bajer A . Cine-micrographic studies on chromosome movements in beta-irradiated cell. Chromosoma. 1958;9:319–31. 10.1007/BF02568084 13608834

[6] McGrath RA, Williams RW. Reconstruction *in vivo* of irradiated *Escherichia coli* deoxyribonucleic acid; the rejoining of broken pieces. Nature. 1966;212:534–5. 10.1038/212534a0 5339143

[7] Ward JF, Kuo I. Radiation damage to DNA in aqueous solution: a comparison of the response of the single-stranded form with that of the double-stranded form. Radiat Res. 1978;75:278–85. 10.2307/3574903 568810

[8] Featherstone C, Jackson SP. DNA double-strand break repair. Curr Biol. 1999;9:R759–61. 10.1016/S0960-9822(00)80005-6 10531043

[9] Schipler A, Iliakis G. DNA double-strand-break complexity levels and their possible contributions to the probability for error-prone processing and repair pathway choice. Nucleic Acids Res. 2013;41:7589–605. 10.1093/nar/gkt556 PMC3763544 23804754

[10] Ngo W, Wu JLY, Wasko KM et al. Targeted delivery of genome editors *in vivo*. Nat Biotechnol. 2026;44:49–59. 10.1038/s41587-025-02945-w PMC12875382 41526513

[11] Bouwman BAM, Crosetto N. Endogenous DNA double-strand breaks during DNA transactions: emerging insights and methods for genome-wide profiling. Genes. 2018;9:632. 10.3390/genes9120632 PMC6316733 30558210

[12] Crosetto N, Mitra A, Silva MJ et al. Nucleotide-resolution DNA double-strand break mapping by next-generation sequencing. Nat Methods. 2013;10:361–5. 10.1038/nmeth.2408 PMC3651036 23503052

[13] Hoang-Bujnowicz A, Wesolowska J, Ostromecka A et al. Critical assessment of microscopy methods for detecting single- and double-strand DNA breaks. Folia Histochem Cytobiol. 2025;63:151–61. 10.5603/fhc.109368 41283282

[14] Nelms BE, Maser RS, MacKay JF et al. *In situ* visualization of DNA double-strand break repair in human fibroblasts. Science. 1998;280:590–2. 10.1126/science.280.5363.590 9554850

[15] Rogakou EP, Boon C, Redon C et al. Megabase chromatin domains involved in DNA double-strand breaks *in vivo*. J Cell Biol. 1999;146:905–16. 10.1083/jcb.146.5.905 PMC2169482 10477747

[16] Gavrieli Y, Sherman Y, Ben-Sasson SA. Identification of programmed cell death *in situ* via specific labeling of nuclear DNA fragmentation. J Cell Biol. 1992;119:493–501. 10.1083/jcb.119.3.493 PMC2289665 1400587

[17] Gorczyca W, Bruno S, Darzynkiewicz R et al. DNA strand breaks occurring during apoptosis - their early *in situ* detection by the terminal deoxynucleotidyl transferase and nick translation assays and prevention by serine protease inhibitors. Int J Oncol. 1992;1:639–48. 10.3892/ijo.1.6.639 21584593

[18] Lutze J, Warrington SE, Kron SJ. TdT-dUTP DSB end labeling (TUDEL), for specific, direct *in situ* labeling of DNA double-strand breaks. Methods Mol Biol. 2022;2394:299–317. 10.1007/978-1-0716-1811-0_18 PMC8820263 35094335

[19] Kordon MM, Zarębski M, Solarczyk K et al. STRIDE-a fluorescence method for direct, specific *in situ* detection of individual single- or double-strand DNA breaks in fixed cells. Nucleic Acids Res. 2020;48:e14. 10.1093/nar/gkz1118 PMC7026605 31832687

[20] Galbiati A, Beauséjour C, Fagagna FdAd. A novel single-cell method provides direct evidence of persistent DNA damage in senescent cells and aged mammalian tissues. Aging Cell. 2017;16:422–7. 10.1111/acel.12573 PMC5334542 28124509

[21] Barski A, Cuddapah S, Cui K et al. High-resolution profiling of histone methylations in the human genome. Cell. 2007;129:823–37. 10.1016/j.cell.2007.05.009 17512414

[22] Johnson DS, Mortazavi A, Myers RM et al. Genome-wide mapping of *in vivo* protein–DNA interactions. Science. 2007;316:1497–502. 10.1126/science.1141319 17540862

[23] Mikkelsen TS, Ku M, Jaffe DB et al. Genome-wide maps of chromatin state in pluripotent and lineage-committed cells. Nature. 2007;448:553–60. 10.1038/nature06008 PMC2921165 17603471

[24] Robertson G, Hirst M, Bainbridge M et al. Genome-wide profiles of STAT1 DNA association using chromatin immunoprecipitation and massively parallel sequencing. Nat Methods. 2007;4:651–7. 10.1038/nmeth1068 17558387

[25] Iacovoni JS, Caron P, Lassadi I et al. High-resolution profiling of gammaH2AX around DNA double-strand breaks in the mammalian genome. EMBO J. 2010;29:1446–57. 10.1038/emboj.2010.38 PMC2868577 20360682

[26] Tsai SQ, Zheng Z, Nguyen NT et al. GUIDE-seq enables genome-wide profiling of off-target cleavage by CRISPR-Cas nucleases. Nat Biotechnol. 2015;33:187–97. 10.1038/nbt.3117 PMC4320685 25513782

[27] Gabriel R, Lombardo A, Arens A et al. An unbiased genome-wide analysis of zinc-finger nuclease specificity. Nat Biotechnol. 2011;29:816–23. 10.1038/nbt.1948 21822255

[28] Klein IA, Resch W, Jankovic M et al. Translocation-capture sequencing reveals the extent and nature of chromosomal rearrangements in B lymphocytes. Cell. 2011;147:95–106. 10.1016/j.cell.2011.07.048 PMC3190307 21962510

[29] Frock RL, Hu J, Meyers RM et al. Genome-wide detection of DNA double-stranded breaks induced by engineered nucleases. Nat Biotechnol. 2015;33:179–86. 10.1038/nbt.3101 PMC4320661 25503383

[30] Hu J, Meyers RM, Dong J et al. Detecting DNA double-stranded breaks in mammalian genomes by linear amplification-mediated high-throughput genome-wide translocation sequencing. Nat Protoc. 2016;11:853–71. 10.1038/nprot.2016.043 PMC4895203 27031497

[31] Rogakou EP, Pilch DR, Orr AH et al. DNA double-stranded breaks induce histone H2AX phosphorylation on serine 139. J Biol Chem. 1998;273:5858–68. 10.1074/jbc.273.10.5858 9488723

[32] Chan DW, Chen BPC, Prithivirajsingh S et al. Autophosphorylation of the DNA-dependent protein kinase catalytic subunit is required for rejoining of DNA double-strand breaks. Genes Dev. 2002;16:2333–8. 10.1101/gad.1015202 PMC187438 12231622

[33] Ying S, Chen Z, Medhurst AL et al. DNA-PKcs and PARP1 bind to unresected stalled DNA replication forks where they recruit XRCC1 to mediate repair. Cancer Res. 2016;76:1078–88. 10.1158/0008-5472.CAN-15-0608 PMC4867494 26603896

[34] Stewart GS, Wang B, Bignell CR et al. MDC1 is a mediator of the mammalian DNA damage checkpoint. Nature. 2003;421:961–6. 10.1038/nature01446 12607005

[35] Bakkenist CJ, Kastan MB. DNA damage activates ATM through intermolecular autophosphorylation and dimer dissociation. Nature. 2003;421:499–506. 10.1038/nature01368 12556884

[36] Schultz LB, Chehab NH, Malikzay A et al. p53 binding protein 1 (53BP1) is an early participant in the cellular response to DNA double-strand breaks. J Cell Biol. 2000;151:1381–90. 10.1083/jcb.151.7.1381 PMC2150674 11134068

[37] Anderson L, Henderson C, Adachi Y. Phosphorylation and rapid relocalization of 53BP1 to nuclear foci upon DNA damage. Mol Cell Biol. 2001;21:1719–29. 10.1128/MCB.21.5.1719-1729.2001 PMC86718 11238909

[38] Li W, Li F, Huang Q et al. Quantitative, noninvasive imaging of radiation-induced DNA double-strand breaks *in vivo*. Cancer Res. 2011;71:4130–7. 10.1158/0008-5472.CAN-10-2540 PMC3117017 21527553

[39] De Waard A, Paul AV, Lehman IR. The structural gene for deoxyribonucleic acid polymerase in bacteriophages T4 and T5. Proc Natl Acad Sci USA. 1965;54:1241–8. 10.1073/pnas.54.4.1241 PMC219850 5219829

[40] Biernacka A, Zhu Y, Skrzypczak M et al. i-BLESS is an ultra-sensitive method for detection of DNA double-strand breaks. Commun Biol. 2018;1:181. 10.1038/s42003-018-0165-9 PMC6208412 30393778

[41] Joshi I, DeRycke J, Palmowski M et al. Genome-wide mapping of DNA double-strand breaks from eukaryotic cell cultures using break-seq. STAR Protocols. 2021;2:100554. 10.1016/j.xpro.2021.100554 PMC8220103 34189468

[42] Hoffman EA, McCulley A, Haarer B et al. Break-seq reveals hydroxyurea-induced chromosome fragility as a result of unscheduled conflict between DNA replication and transcription. Genome Res. 2015;25:402–12. 10.1101/gr.180497.114 PMC4352882 25609572

[43] Canela A, Sridharan S, Sciascia N et al. DNA breaks and end resection measured genome-wide by end sequencing. Mol Cell. 2016;63:898–911. 10.1016/j.molcel.2016.06.034 PMC6299834 27477910

[44] Wong N, John S, Nussenzweig A et al. END-seq: an unbiased, high-resolution, and genome-wide approach to map DNA double-strand breaks and resection in human cells. Methods Mol Biol. 2021;2153:9–31. 10.1007/978-1-0716-0644-5_2 32840769

[45] Mitra A, Skrzypczak M, Ginalski K et al. Strategies for achieving high sequencing accuracy for low diversity samples and avoiding sample bleeding using illumina platform. PLoS One. 2015;10:e0120520. 10.1371/journal.pone.0120520 PMC4393298 25860802

[46] Lensing SV, Marsico G, Hänsel-Hertsch R et al. DSBCapture: *in situ* capture and sequencing of DNA breaks. Nat Methods. 2016;13:855–7. 10.1038/nmeth.3960 PMC5045719 27525976

[47] Yan WX, Mirzazadeh R, Garnerone S et al. BLISS is a versatile and quantitative method for genome-wide profiling of DNA double-strand breaks. Nat Commun. 2017;8:15058. 10.1038/ncomms15058 PMC5437291 28497783

[48] Biernacka A, Skrzypczak M, Zhu Y et al. High-resolution, ultrasensitive and quantitative DNA double-strand break labeling in eukaryotic cells using i-BLESS. Nat Protoc. 2021;16:1034–61. 10.1038/s41596-020-00448-3 PMC8088906 33349705

[49] Zhu Y, Biernacka A, Pardo B et al. qDSB-Seq is a general method for genome-wide quantification of DNA double-strand breaks using sequencing. Nat Commun. 2019;10:2313. 10.1038/s41467-019-10332-8 PMC6534554 31127121

[50] Bouwman BAM, Agostini F, Garnerone S et al. Genome-wide detection of DNA double-strand breaks by in-suspension BLISS. Nat Protoc. 2020;15:3894–941. 10.1038/s41596-020-0397-2 33139954

[51] Dobbs FM, Eijk Pv, Fellows MD et al. Precision digital mapping of endogenous and induced genomic DNA breaks by INDUCE-seq. Nat Commun. 2022;13:3989. 10.1038/s41467-022-31702-9 PMC9271039 35810156

[52] Chiarle R, Zhang Y, Frock RL et al. Genome-wide translocation sequencing reveals mechanisms of chromosome breaks and rearrangements in B cells. Cell. 2011;147:107–19. 10.1016/j.cell.2011.07.049 PMC3186939 21962511

[53] Shee C, Cox BD, Gu F et al. Engineered proteins detect spontaneous DNA breakage in human and bacterial cells. eLife. 2013;2:e01222. 10.7554/eLife.01222 PMC3809393 24171103

[54] Schimmel J, Kool H, Schendel Rv et al. Mutational signatures of non-homologous and polymerase theta-mediated end-joining in embryonic stem cells. EMBO J. 2017;36:3634–49. 10.15252/embj.201796948 PMC5730883 29079701

[55] Deshpande S, Yang Y, Chilkoti A et al. Enzymatic synthesis and modification of high molecular weight DNA using terminal deoxynucleotidyl transferase. Methods Enzymol. 2019;627:163–88. 10.1016/bs.mie.2019.07.044 PMC7241426 31630739

[56] Burma S, Chen BP, Murphy M et al. ATM phosphorylates histone H2AX in response to DNA double-strand breaks. J Biol Chem. 2001;276:42462–7. 10.1074/jbc.C100466200 11571274

[57] Conic S, Desplancq D, Ferrand A et al. Imaging of native transcription factors and histone phosphorylation at high resolution in live cells. J Cell Biol. 2018;217:1537–52. 10.1083/jcb.201709153 PMC5881509 29440513

[58] Trakarnphornsombat W, Kimura H. Live-cell tracking of γ-H2AX kinetics reveals the distinct modes of ATM and DNA-PK in the immediate response to DNA damage. J Cell Sci. 2023;136:jcs260698. 10.1242/jcs.260698 PMC10163350 36999484

[59] Moeglin E, Desplancq D, Stoessel A et al. A novel nanobody precisely visualizes phosphorylated histone H2AX in living cancer cells under drug-induced replication stress. Cancers. 2021;13:3317. 10.3390/cancers13133317 PMC8267817 34282773

[60] Mirzoeva OK, Petrini JH. DNA damage-dependent nuclear dynamics of the Mre11 complex. Mol Cell Biol. 2001;21:281–8. 10.1128/MCB.21.1.281-288.2001 PMC88801 11113202

[61] Chapman JR, Sossick AJ, Boulton SJ et al. BRCA1-associated exclusion of 53BP1 from DNA damage sites underlies temporal control of DNA repair. J Cell Sci. 2012;125:3529–34. 10.1242/jcs.105353 PMC3445322 22553214

[62] Feng L, Li N, Li Y et al. Cell cycle-dependent inhibition of 53BP1 signaling by BRCA1. Cell Discov. 2015;1:15019. 10.1038/celldisc.2015.19 PMC4860855 27462418

[63] Schwarz B, Friedl AA, Girst S et al. Nanoscopic analysis of 53BP1, BRCA1 and Rad51 reveals new insights in temporal progression of DNA-repair and pathway choice. Mutat Res. 2019;816–818:111675. 10.1016/j.mrfmmm.2019.111675 31302572

[64] Mohapatra S, Kawahara M, Khan IS et al. Restoration of G1 chemo/radioresistance and double-strand-break repair proficiency by wild-type but not endonuclease-deficient Artemis. Nucleic Acids Res. 2011;39:6500–10. 10.1093/nar/gkr257 PMC3159448 21531702

[65] Ahnesorg P, Smith P, Jackson SP. XLF interacts with the XRCC4-DNA ligase IV complex to promote DNA nonhomologous end-joining. Cell. 2006;124:301–13. 10.1016/j.cell.2005.12.031 16439205

[66] Balajee AS, Geard CR. Replication protein A and gamma-H2AX foci assembly is triggered by cellular response to DNA double-strand breaks. Exp Cell Res. 2004;300:320–34. 10.1016/j.yexcr.2004.07.022 15474997

[67] Bhattacharya S, Srinivasan K, Abdisalaam S et al. RAD51 interconnects between DNA replication, DNA repair and immunity. Nucleic Acids Res. 2017;45:4590–605. 10.1093/nar/gkx126 PMC5416901 28334891

[68] Scully R, Chen J, Ochs RL et al. Dynamic changes of BRCA1 subnuclear location and phosphorylation state are initiated by DNA damage. Cell. 1997;90:425–35. 10.1016/S0092-8674(00)80503-6 9267023

[69] Panier S, Boulton SJ. Double-strand break repair: 53BP1 comes into focus. Nat Rev Mol Cell Biol. 2014;15:7–18. 10.1038/nrm3719 24326623

[70] Ward IM, Minn K, Jorda KG et al. Accumulation of checkpoint protein 53BP1 at DNA breaks involves its binding to phosphorylated histone H2AX. J Biol Chem. 2003;278:19579–82. 10.1074/jbc.C300117200 12697768

[71] Kelliher JL, Folkerts ML, Shen KV et al. Evolved histone tail regulates 53BP1 recruitment at damaged chromatin. Nat Commun. 2024;15:4634. 10.1038/s41467-024-49071-w PMC11143218 38821984

[72] Mirman Z, Lange Td. 53BP1: a DSB escort. Genes Dev. 2020;34:7–23. 10.1101/gad.333237.119 PMC6938671 31896689

[73] Rybak P, Hoang A, Bujnowicz L et al. Low level phosphorylation of histone H2AX on serine 139 (γH2AX) is not associated with DNA double-strand breaks. Oncotarget. 2016;7:49574–87. 10.18632/oncotarget.10411 PMC5226530 27391338

[74] Cleaver JE . γH2Ax: biomarker of damage or functional participant in DNA repair ‘all that glitters is not gold!’. Photochem Photobiol. 2011;87:1230–9. 10.1111/j.1751-1097.2011.00995.x 21883247

[75] Richardson CC . Phosphorylation of nucleic acid by an enzyme from T4 bacteriophage-infected *Escherichia coli*. Proc Natl Acad Sci USA. 1965;54:158–65. 10.1073/pnas.54.1.158 PMC285814 5323016

[76] Marková E, Schultz N, Belyaev IY. Kinetics and dose-response of residual 53BP1/gamma-H2AX foci: co-localization, relationship with DSB repair and clonogenic survival. Int J Radiat Biol. 2007;83:319–29. 10.1080/09553000601170469 17457757

[77] Clouaire T, Rocher V, Lashgari A et al. Comprehensive mapping of histone modifications at DNA double-strand breaks deciphers repair pathway chromatin signatures. Mol Cell. 2018;72:250–62.e6. 10.1016/j.molcel.2018.08.020 PMC6202423 30270107

[78] Iannelli F, Galbiati A, Capozzo I et al. A damaged genome’s transcriptional landscape through multilayered expression profiling around *in situ*-mapped DNA double-strand breaks. Nat Commun. 2017;8:15656. 10.1038/ncomms15656 PMC5499205 28561034

[79] Caron P, Choudjaye J, Clouaire T et al. Non-redundant functions of ATM and DNA-PKcs in response to DNA double-strand breaks. Cell Rep. 2015;13:1598–609. 10.1016/j.celrep.2015.10.024 PMC4670905 26586426

[80] Dobbs F . Determining the mechanism of off-target mutagenesis caused by CRISPR-Cas9 genome editing. Phd, Cardiff University, 2021. https://orca.cardiff.ac.uk/id/eprint/143377/ (16 June 2026, date last accessed).

[81] Gorczyca W, Gong J, Darzynkiewicz Z. Detection of DNA strand breaks in individual apoptotic cells by the *in situ* terminal deoxynucleotidyl transferase and nick translation assays. Cancer Res. 1993;53:1945–51. 8467513

[82] Collins AR, Oscoz AA, Brunborg G et al. The comet assay: topical issues. Mutagenesis. 2008;23:143–51. 10.1093/mutage/gem051 18283046

[83] Tu WZ, Li B, Huang B et al. γH2AX foci formation in the absence of DNA damage: mitotic H2AX phosphorylation is mediated by the DNA-PKcs/CHK2 pathway. FEBS Lett. 2013;587:3437–43. 10.1016/j.febslet.2013.08.028 24021642

[84] Nakamura AJ, Rao VA, Pommier Y et al. The complexity of phosphorylated H2AX foci formation and DNA repair assembly at DNA double-strand breaks. Cell Cycle. 2010;9:389–97. 10.4161/cc.9.2.10475 PMC3086803 20046100

[85] Gouge J, Rosario S, Romain F et al. Structural basis for a novel mechanism of DNA bridging and alignment in eukaryotic DSB DNA repair. EMBO J. 2015;34:1126–42. 10.15252/embj.201489643 PMC4406656 25762590

[86] Alsemarz A, Lasko P, Fagotto F. Limited significance of the *in situ* proximity ligation assay. bioRxiv, 10.1101/411355, 5 November 2018, preprint: not peer reviewed.

[87] Solarczyk KJ, Zarębski M, Dobrucki JW. Inducing local DNA damage by visible light to study chromatin repair. DNA Repair. 2012;11:996–1002. 10.1016/j.dnarep.2012.09.008 23089313

[88] D’Abrantes S, Gratton S, Reynolds P et al. Super-resolution nanoscopy imaging applied to DNA double-strand breaks. Radiat Res. 2018;189:19–31. 10.1667/RR14594.1 29053406

[89] Martín M, Terradas M, Hernández L et al. γH2AX foci on apparently intact mitotic chromosomes: not signatures of misrejoining events but signals of unresolved DNA damage. Cell Cycle. 2014;13:3026–36. 10.4161/15384101.2014.947786 PMC4614418 25486563

[90] Zarębski M, Bosire R, Wesołowska J et al. Translocation of chromatin proteins to nucleoli-The influence of protein dynamics on post-fixation localization. Cytometry A. 2021;99:1230–9. 10.1002/cyto.a.24464 PMC9543561 34110091

[91] Richter KN, Revelo NH, Seitz KJ et al. Glyoxal as an alternative fixative to formaldehyde in immunostaining and super-resolution microscopy. EMBO J. 2018;37:139–59. 10.15252/embj.201695709 PMC5753035 29146773

[92] Heyza JR, Mikhova M, Bahl A et al. Systematic analysis of the molecular and biophysical properties of key DNA damage response factors. eLife. 2023;12:e87086. 10.7554/eLife.87086 PMC10319438 37341699

[93] Mok MTS, Henderson BR. The *in vivo* dynamic interplay of MDC1 and 53BP1 at DNA damage-induced nuclear foci. Int J Biochem Cell Biol. 2012;44:1398–409. 10.1016/j.biocel.2012.05.025 22677490

[94] Modica G, Roy J, Godin AG et al. Focus on numbers - characterizing protein accumulation at DNA double-strand breaks. J Cell Sci. 2025;138:jcs263483. 10.1242/jcs.263483 40309929

[95] Heyza JR, Mikhova M, Schmidt JC. Live cell single-molecule imaging to study DNA repair in human cells. DNA Repair. 2023;129:103540. 10.1016/j.dnarep.2023.103540 PMC10530516 37467632

[96] Levchenko SM, Dobrucki JW. High molecular crowding in repair foci surrounding DNA breaks, measured by fluorescence lifetime imaging microscopy. FASEB J. 2025;39:e71010. 10.1096/fj.202501727R PMC12404230 40892367

[97] Milo R . What is the total number of protein molecules per cell volume? A call to rethink some published values. Bioessays. 2013;35:1050–5. 10.1002/bies.201300066 PMC3910158 24114984

[98] Sax K . Chromosome aberrations induced by X-Rays. Genetics. 1938;23:494–516. 10.1093/genetics/23.5.494 PMC1209022 17246897

[99] Vilenchik MM, Knudson AG. Endogenous DNA double-strand breaks: production, fidelity of repair, and induction of cancer. Proc Natl Acad Sci USA. 2003;100:12871–6. 10.1073/pnas.2135498100 PMC240711 14566050

[100] Suh EK, Yang A, Kettenbach A et al. p63 protects the female germ line during meiotic arrest. Nature. 2006;444:624–8. 10.1038/nature05337 17122775

[101] Zhu Y, Biernacka A, Pardo B et al. Integrated analysis of patterns of DNA breaks reveals break formation mechanisms and their population distribution during replication stress. bioRxiv, 10.1101/171439, 18 September 2017, preprint: not peer reviewed.

[102] Luca KLd, Rullens PMJ, Karpinska MA et al. Genome-wide profiling of DNA repair proteins in single cells. Nat Commun. 2024;15:9918. 10.1038/s41467-024-54159-4 PMC11582664 39572529

[103] Warters RL, Adamson PJ, Pond CD et al. Melanoma cells express elevated levels of phosphorylated histone H2AX foci. J Invest Dermatol. 2005;124:807–17. 10.1111/j.0022-202X.2005.23674.x 15816840

[104] Juan G, Zoog SJ, Ferbas J. Leveraging image cytometry for the development of clinically feasible biomarkers: evaluation of activated caspase-3 in fine needle aspirate biopsies. Methods Cell Biol. 2011;102:309–20. 10.1016/B978-0-12-374912-3.00012-2 21704844

[105] Dugle DL, Gillespie CJ, Chapman JD. DNA strand breaks, repair, and survival in x-irradiated mammalian cells. Proc Natl Acad Sci USA. 1976;73:809–12. 10.1073/pnas.73.3.809 PMC336008 1062792

[106] Pan J, Sasaki M, Kniewel R et al. A hierarchical combination of factors shapes the genome-wide topography of yeast meiotic recombination initiation. Cell. 2011;144:719–31. 10.1016/j.cell.2011.02.009 PMC3063416 21376234

[107] Dang W, Sutphin GL, Dorsey JA et al. Inactivation of yeast Isw2 chromatin remodeling enzyme mimics longevity effect of calorie restriction via induction of genotoxic stress response. Cell Metab. 2014;19:952–66. 10.1016/j.cmet.2014.04.004 PMC4106248 24814484

[108] Hsu PD, Lander ES, Zhang F. Development and applications of CRISPR-Cas9 for genome engineering. Cell. 2014;157:1262–78. 10.1016/j.cell.2014.05.010 PMC4343198 24906146

[109] Carroll D . Genome engineering with zinc-finger nucleases. Genetics. 2011;188:773–82. 10.1534/genetics.111.131433 PMC3176093 21828278

[110] Joung JK, Sander JD. TALENs: a widely applicable technology for targeted genome editing. Nat Rev Mol Cell Biol. 2013;14:49–55. 10.1038/nrm3486 PMC3547402 23169466

[111] Tycko J, Myer VE, Hsu PD. Methods for optimizing CRISPR-Cas9 genome editing specificity. Mol Cell. 2016;63:355–70. 10.1016/j.molcel.2016.07.004 PMC4976696 27494557

[112] Lek A, Wong B, Keeler A et al. Death after high-dose rAAV9 gene therapy in a patient with Duchenne’s muscular dystrophy. N Engl J Med. 2023;389:1203–10. 10.1056/NEJMoa2307798 PMC11288170 37754285

[113] Kaiser J . Gene therapy trials for sickle cell disease halted after two patients develop cancer. Science. 2021; published online 16 Feb. https://www.science.org/content/article/gene-therapy-trials-sickle-cell-disease-halted-after-two-patients-develop-cancer

[114] Servick K . Gene therapy clinical trial halted as cancer risk surfaces. Science. 2021; published online 11 August. https://www.science.org/content/article/gene-therapy-clinical-trial-halted-cancer-risk-surfaces

[115] Cheng Y, Tsai SQ. Illuminating the genome-wide activity of genome editors for safe and effective therapeutics. Genome Biol. 2018;19:226. 10.1186/s13059-018-1610-2 PMC6303961 30577870

[116] Akcakaya P, Bobbin ML, Guo JA et al. *In vivo* CRISPR editing with no detectable genome-wide off-target mutations. Nature. 2018;561:416–9. 10.1038/s41586-018-0500-9 PMC6194229 30209390

[117] FDA . Human gene therapy products incorporating human genome editing. 8 September 2024. https://www.fda.gov/regulatory-information/search-fda-guidance-documents/human-gene-therapy-products-incorporating-human-genome-editing (2 July 2026, date last accessed).

[118] Skeens E, Sinha S, Ahsan M et al. High-fidelity, hyper-accurate, and evolved mutants rewire atomic-level communication in CRISPR-Cas9. Sci Adv. 2024;10:eadl1045. 10.1126/sciadv.adl1045 PMC10917355 38446895

